# Juvenile rockfish show resilience to CO_2_-acidification and hypoxia across multiple biological scales

**DOI:** 10.1093/conphys/coy038

**Published:** 2018-07-10

**Authors:** Brittany E Davis, Lisa M Komoroske, Matthew J Hansen, Jamilynn B Poletto, Emily N Perry, Nathan A Miller, Sean M Ehlman, Sarah G Wheeler, Andrew Sih, Anne E Todgham, Nann A Fangue

**Affiliations:** 1 Department of Wildlife, Fish and Conservation Biology, University of California Davis, Davis, CA, USA; 2 Department of Animal Sciences, University of California Davis, Davis, CA, USA; 3 Bodega Marine Laboratory, University of California Davis, Bodega Bay, CA, USA; 4 Department of Environmental Conservation, University of Massachusetts Amherst, Amherst, MA, USA; 5 School of Natural Resources, University of Nebraska, Lincoln, NE, USA; 6 Department of Environmental Science and Policy, University of California Davis, Davis, CA, USA; 7 Department of Biology, San Diego State University, San Diego, CA, USA

**Keywords:** Anti-predator behavior, cabezon, climate change, physiology, *Sebastes*

## Abstract

California’s coastal ecosystems are forecasted to undergo shifting ocean conditions due to climate change, some of which may negatively impact recreational and commercial fish populations. To understand if fish populations have the capacity to respond to multiple stressors, it is critical to examine interactive effects across multiple biological scales, from cellular metabolism to species interactions. This study examined the effects of CO_2_-acidification and hypoxia on two naturally co-occurring species, juvenile rockfish (genus *Sebastes)* and a known predator, cabezon (*Scorpaenichthys marmoratus*). Fishes were exposed to two *P*CO_2_ levels at two dissolved oxygen (DO) levels: ~600 (ambient) and ~1600 (high) μatm *P*CO_2_ and 8.0 (normoxic) and 4.5 mg l^−1^ DO (hypoxic) and assessments of cellular metabolism, prey behavior and predation mortality rates were quantified after 1 and 3 weeks. Physiologically, rockfish showed acute alterations in cellular metabolic enzyme activity after 1 week of acclimation to elevated *P*CO_2_ and hypoxia that were not evident in cabezon. Alterations in rockfish energy metabolism were driven by increases in anaerobic LDH activity, and adjustments in enzyme activity ratios of cytochrome c oxidase and citrate synthase and LDH:CS. Correlated changes in rockfish behavior were also apparent after 1 week of acclimation to elevated *P*CO_2_ and hypoxia. Exploration behavior increased in rockfish exposed to elevated *P*CO_2_ and spatial analysis of activity indicated short-term interference with anti-predator responses. Predation rate after 1 week increased with elevated *P*CO_2_; however, no mortality was observed under the multiple-stressor treatment suggesting negative effects on cabezon predators. Most noteworthy, metabolic and behavioral changes were moderately compensated after 3 weeks of acclimation, and predation mortality rates also decreased suggesting that these rockfish may be resilient to changes in environmental stressors predicted by climate models. Linking physiological and behavioral responses to multiple stressors is vital to understand impacts on populations and community dynamics.

## Teaser

The present study showed short-term elevations in cellular metabolism, alterations in behavior and susceptibility to predation in juvenile rockfish after acute acclimation to CO_2_-acidification and hypoxic conditions. Physiological and behavioral alterations were restored after 3 weeks suggesting that rockfish possess mechanisms to defend rapid changes in *P*CO_2_ and oxygen conditions.

## Introduction

Climate change is predicted to have profound implications for marine ecosystems, with adverse ramifications expected for species abundance and diversity, and the sustainability of commercial fisheries (Brander, 2007; Wood and McDonald, 1997). Shifts in multiple environmental factors such as elevated dissolved carbon dioxide (CO_2_, hereafter CO_2_-acidification), low dissolved oxygen (DO, hereafter hypoxia) and increased temperature (Crain *et al.*, 2008; Keeling *et al.*, 2010; Doney *et al.*, 2011) have the potential to threaten individual species and communities by negatively affecting animal physiology and behavior (see reviews Heuer and Grosell, 2014; Nagelkerken and Munday, 2016; Tresguerres and Hamilton, 2017). While the organismal responses to CO_2_-acidification and hypoxia have individually been explored, in nature these factors can change in concert. In particular, in highly productive, mid-latitude coastal systems with seasonal upwelling, seawater can be both high in partial pressure of CO_2_ (*P*CO_2_) and low in DO due to expansion of oxygen minimum zones (Chan *et al.*, 2008; Largier *et al.*, 2015). This results in dynamic changes in both pH and oxygen in coastal habitats (Feely *et al.*, 2008).

Environmental stressors can directly impact the physiology of individual animals (Hochachka and Somero, 2002), and physiology is the mechanistic driver of an animal’s behavior (Kane *et al.*, 2005). The effects of abiotic environmental stressors on organismal physiology and behavior can then lead to alterations in trophic interactions, highlighting the need to scale up responses to understand ecological implications of environmental change. To accurately explore the effects of multiple stressors (e.g. CO_2_-acidification and hypoxia), and link organismal mechanisms to community function, it is vital to examine interactive effects across multiple biological scales, from cellular metabolism to species interactions. For example, physiological costs that result in reduced growth or altered locomotion may increase predation risk (Briffa *et al.*, 2012; Allan *et al.*, 2013; Miller *et al.*, 2016). This is a process through which both physiological and behavioral impacts can have fitness consequences and influence population and community dynamics (Hofmann *et al.*, 2010; Munday *et al.*, 2013).

Fish are acid–base regulators, and hence, the effect of CO_2_-acidification on the physiology and behavior of fishes has been projected to be less severe than on other marine species (Kroeker *et al.*, 2010; Heuer and Grosell, 2014). While adult fish are generally less sensitive to CO_2_-acidified seawater, there is accumulating evidence to suggest negative physiological and behavioral costs on larval and juvenile life stages (Munday *et al.*, 2009; Ferrari *et al.*, 2011a, b, 2012; Simpson *et al.*, 2011; Baumann *et al.*, 2012; Allan *et al.*, 2013; Chivers *et al.*, 2014; Chung *et al.*, 2014; Davis *et al.*, 2017). Early life stages of fish have also been shown to be sensitive to hypoxia (Pichavant *et al.*, 2000; Taylor and Miller, 2001; Depasquale *et al.*, 2015). Juvenile fish energetic demands are driven by development and growth such that multiple environmental stressors may create trade-offs, both physiological (e.g. energy allocation for stress response mechanisms and growth and metabolism [Pörtner *et al.*, 2005; Ishimatsu *et al.*, 2008; Pankhurst and Munday, 2011; Sokolova *et al.*, 2012]) and behavioral (e.g. foraging activity and avoiding predation [Lima and Dill, 1990; Houston *et al.*, 1993]). Specifically, CO_2_-acidification and hypoxia can exert sub-lethal physiological impacts that influence performance of animals (Ishimatsu *et al.*, 2004; Sokolova, 2013; Todgham and Stillman, 2013; Steckbauer *et al.*, 2015; Depasquale *et al.*, 2015; Gobler and Baumann, 2016) through increased energetic costs (Hamilton *et al.*, 2017) or a heightened stress response (Hamilton *et al.*, 2014). It is also likely that many species of juvenile fish have a lower capacity to behaviorally avoid changing conditions compared to adult fishes, as early life stages are often more constrained to particular microhabitats due to interactions between abiotic factors and predation risk (Blaber and Blaber, 1980; Laegdsgaard and Johnson, 2001). Therefore, with an inability to avoid adverse environmental conditions, stressors may cause suboptimal performance resulting in slower growth (Gobler *et al.*, 2014; Depasquale *et al.*, 2015), reduced feeding (Appelhans *et al.*, 2012; Thorarensen *et al.*, 2017) and an increased susceptibility to predation (Briffa *et al.*, 2012; Allan *et al.*, 2013; Miller *et al.*, 2016).

Although both predators and prey in nature will likely be exposed to similar environments, most studies to date have only exposed prey species to various projected environmental changes and then tested their capacity to evade predation by predators that were held under control conditions (but see Ferrari *et al.*, 2015). However, if predation efficiency is also impacted by CO_2_-acidification or hypoxia, predator–prey dynamics would be very different than outcomes from studies where only the prey has been exposed. Therefore, the impacts of CO_2_-acidification and hypoxia on biotic interactions can be more robustly evaluated with multi-species exposure studies and will provide insights for community-level impacts if multiple stressor impacts are mediated through food webs (Pörtner and Peck, 2010).

Fishes living in seagrass habitats are regularly exposed to highly variable pH and DO, due to respiration and photosynthesis in tandem with other factors such as upwelling (Diaz and Rosenberg, 2008; Diaz and Breitburg, 2009; Torres *et al.*, 2012); however, these habitats are often overlooked when examining the influence of environmental stressors on organismal physiology and behavior. Previous studies on closely related juvenile rockfish (genus *Sebastes*) showed that physiological and behavioral responses to elevated *P*CO_2_ were dependent on life-history strategies (Hamilton *et al.*, 2017). For example, juvenile blue rockfish (*S. mystinus*) that inhabit deeper, low pH habitats were relatively robust to laboratory-elevated *P*CO_2_ conditions. In contrast, juvenile copper rockfish (*S. caurinus*) that inhabit shallower-surface waters of kelp forests with dynamic fluctuations in pH (and DO levels) showed increased sensitivities to elevated *P*CO_2_ exposures, with alterations in swimming, metabolism and gene expression (Hamilton *et al.*, 2017). To date, physiological and behavioral responses to elevated *P*CO_2_ and hypoxia of juvenile rockfish (genus *Sebastes*) that develop in a highly fluctuating, variable environment such as seagrass beds have not been explored. Rockfish that inhabit seagrass beds may be particularly robust to environmental perturbation with an enhanced ability to compensate for alterations in *P*CO_2_ and DO compared to species living in more stable environments. Additionally, no studies have assessed the effects of multiple stressors on the interactions between juvenile rockfish and their predators, juvenile cabezon (*Scorpaenichthys marmoratus*) that share the same fluctuating *P*CO_2_ and DO environment.

Juvenile rockfish and cabezon provide a good study system to explore the interacting effects of multiple environmental stressors (CO_2_-acidification and hypoxia) at multiple biological scales (i.e. cellular physiology and predator-prey dynamics), and to assess whether these fishes have an ability to compensate physiologically or behaviorally to these stressors. The objectives of this study were therefore; (1) to determine if there are energetic costs associated with acute (1 week) and chronic (3 week) exposures to elevated *P*CO_2_ and low DO (hypoxic) conditions in juvenile rockfish and cabezon, (2) to characterize alterations in rockfish movement and antipredator behaviors in response to alarm cue exposure, and finally (3) to assess if actual predator–prey relationships may be altered under exposures to high *P*CO_2_ and hypoxic environments. We predicted that there would be alterations in aerobic and anaerobic metabolic pathways that would affect the capacity to generate ATP after 1 and 3 weeks of acclimation to the two stressors (Windisch *et al.*, 2011; Hamilton *et al.*, 2017), and that movement behavior would also be altered to conserve energy (Herbert and Steffensen, 2005; Davis *et al.*, 2017). Predictions for alterations in behavior, and in turn, effects on predation rates may not be as straight forward with potential interactions between multiple stressors. Elevated *P*CO_2_ may reduce anti-predator responses (Ferrari *et al.*, 2012), but hypoxia might also reduce activity which tends to reduce encounter rates with predators. Additionally, either high *P*CO_2_ or hypoxia might reduce swimming and escape ability in rockfish, but also have negative effects on predator activity or attack ability. Therefore, overall effects on predation rates of rockfish under multiple stressors of high *P*CO_2_ and hypoxia are likely complex. By examining the effects of multiple stressors across biological scales of two naturally-interacting species, this study will help our understanding of potential ‘trade-offs’ in physiological mechanisms and behavioral strategies of prey, and how that might alter predator–prey dynamics of two recreationally and commercially important species (Parker *et al.*, 2000).

## Materials and methods

### Fish collection and maintenance

Juvenile rockfish (*Sebastes spp*.) and cabezon (*S. marmoratus*) were collected from seagrass beds in Campbell Cove, CA, USA (38°18′19″N, 123°03′28″W) using beach seine nets. Rockfish and cabezon were collected twice for two successive experiments. Fish for the first physiology experiment (Phase I) were collected late April–May 2015 (rockfish: 28.96 ± 2.54 mm and 0.41 ± 0.14 g and cabezon: 83.92 ± 10.97 mm and 12.69 ± 5.44 g, mean ± SD); whereas fish for the second behavior and predation experiment (Phase II) were collected July–August 2015 (rockfish: 31.81 ± 3.18 mm and 0.71 ± 0.22 g and cabezon: 83.21 ± 10.72 mm and 13.77 ± 4.60 g, mean ± SD). Fish were immediately transported to the Bodega Marine Laboratory, University of California Davis, within 2 h of collection. Rockfish and cabezon were held in separate flow-through seawater tanks (32 ± 0.1 ppt, 14°C ± 1°C) for four (Phase II) or six (Phase I) weeks until the start of experiments. Fish were fed finely chopped frozen squid *ad libitum* daily during the laboratory acclimation period. This research project was conducted in accordance with animal welfare laws approved by the University of California Davis Institutional Animal Care and Use Committee.

All experiments were conducted at the subgenera level, using *Pteropodus* rockfishes. Post-experimental DNA barcoding of the cytochrome c oxidase loci (COI) conducted on only a small subset of rockfish confirmed that the study included a mix of species in the complex of seagrass rockfishes including gopher rockfish (*S. caratus*), copper rockfish (*S. caurinus*), and black-and-yellow rockfish (*S. chrysomelas*), which all exhibit similar ecological and genetic traits. The genetic similarities of rockfish collected in this study made it impossible to DNA barcode rockfish that were consumed by cabezon; however, given the shared ecological and genetic traits of these species (Echeverria, 1987; Love *et al.*, 2002; Li *et al.*, 2006; Wheeler *et al.*, 2017), we assume negligible differences in the physiological and behavioral responses of *Pteropodus* rockfishes. For these reasons, and to maintain consistency with other studies (Caselle *et al.*, 2010a, b; Jones and Mulligan, 2014; Wheeler *et al.*, 2017), all experiments were conducted at the subgenera level (*Pteropodus*).

### Experimental conditions

After four (Phase II) and six (Phase I) weeks of laboratory acclimation, rockfish and cabezon were divided among four *P*CO_2_/DO treatments (two *P*CO_2_ levels × two DO levels) and exposed for a three-week period to assess both main and interactive effects of two environmental factors that change during a typical upwelling event. CO_2_-acidified treatments consisted of two levels, one representing *Ambient P*CO_2_ conditions typically experienced off the California coastline (600 μatm *P*CO_2_), and a second based on upwelling conditions and predicted *High P*CO_2_ levels for the coast (1600 μatm *P*CO_2_). The ambient *P*CO_2_-treatment was selected based on data from Central and Northern California Ocean Observing System (CeNCOOS) showing that ambient *P*CO_2_ levels along Bodega Bay, CA can remain relatively high compared to other areas along the coast (450–650 μatm *P*CO_2_), with spikes above 700 μatm, whereas the predicted High *P*CO_2_ levels were selected based on the worst-case scenario Representative Concentration Pathway (RCP8.5) for climate change (IPCC, 2013). DO treatments included *Normoxic* (8 mg l^−1^ O_2_) and moderately *Hypoxic* (4.5 mg l^−1^ O_2_) conditions consistent with nearshore waters off the coast of California during upwelling (Frieder *et al.*, 2012; Largier *et al.*, 2015; data downloaded from Bodega Ocean Observing Node (BOON) at http://boon.ucdavis.edu.). Each *P*CO_2_/DO treatment combination was attained using a *P*CO_2_, air and N_2_ gas-delivery system (see details in Supplementary Fig. S1; modified from Fangue *et al.*, 2010). Mixed gas was continuously delivered to four 150-l reservoir containers (one per *P*CO_2_/DO treatment) where it was mixed vigorously with seawater until equilibrated. Reservoir *P*CO_2_/DO treatment water was continuously dripped at a rate of 16 l h^−1^ into three 38-l replicate tanks (per treatment) with *n* = 25 rockfish in each. For cabezon, *P*CO_2_/DO treatments were replicated twice with *n* = 4 fish in each 38-l replicate tank. In addition, *P*CO_2_/DO treatment gas was directly bubbled into each replicate tank. All fishes were measured for mass and standard length prior to being put in each tank to acquire a baseline body condition (estimated by Fulton’s K; 100*[mass in g/standard length in cm^3^]). During the experiment, rockfish tanks were fed 1 g of frozen, finely chopped squid daily, whereas cabezon tanks were fed 1 g every other day. All tanks were surrounded by opaque blinds and with separate flow-through, circulating water (16 l h^−1^) to ensure rockfish received no olfactory or visual cues from the cabezon.

### Experimental seawater chemistry

Temperature (°C), salinity (ppt), and DO (mg l^−1^) of each replicate tank for rockfish and cabezon were recorded once daily using a handheld YSI handheld metre (YSI 85, Yellow Springs, OH USA). Total pH was measured from each replicate tank and *P*CO_2_/DO gas mixing reservoir container once every 48 h using m-cresol dye (Sigma-Aldrich, St. Louis, MO, USA) in a UV spectrophotometer (SOP 6b, Shimadzu, Columbia, MD, USA). Every four days, total alkalinity was measured using open-cell titration (T50 titrator, Mettler Toledo Inc., Columbus, OH, USA) of the *P*CO_2_/DO gas mixing reservoir containers (titrant was acquired from the Dickson Laboratory, Scripps Institute, La Jolla, CA, USA). Salinity was additionally measured in the titration samples using a conductivity metre (YSI 3100, Yellow Springs, OH, USA). *P*CO_2_ values were calculated for each replicate tank using temperature, salinity, total pH and alkalinity in R (R Development Core Team, 2013; package *seacarb*, Gattuso *et al.*, 2015) and are summarized in Table 1 for each experimental phase.

**Table 1:** Seawater chemistry maintained for the duration of the experiments. Cellular metabolism data were collected during Phase I, and behavior and predation data were collected during Phase II. Values are presented as mean ± SD

Treatment and species	pH (Total scale)	*P*CO_2_ (μatm)	DO (mg L^−1^)	Alkalinity (μmol kg^−1^)	Temperature (°C)	Salinity (ppt)
Phase I (Physiology)						
*Ambient PCO*_*2*_*+ Normoxia*						
Rockfish	7.86 ± 0.03	669 ± 64	7.6 ± 0.1	2238.9 ± 12.9	13.9 ± 0.1	32.6 ± 0.1
Cabezon	7.81 ± 0.01	744 ± 9	7.3 ± 0.2	2239.3 ± 12.8	13.9 ± 0.1	32.6 ± 0.2
*Ambient PCO*_*2*_*+ Hypoxia*						
Rockfish	7.89 ± 0.01	606 ± 13	4.7 ± 0.2	2238.9 ± 12.4	13.9 ± 0.1	32.6 ± 0.1
Cabezon	7.80 ± 0.02	757 ± 36	4.6 ± 0.1	2239.4 ± 12.3	13.8 ± 0.1	32.6 ± 0.1
*High PCO*_*2*_*+ Normoxia*						
Rockfish	7.50 ± 0.03	1593 ± 114	7.6 ± 0.1	2238.2 ± 12.7	14.0 ± 0.2	32.6 ± 0.1
Cabezon	7.45 ± 0.03	1804 ± 106	7.0 ± 0.3	2237.8 ± 12.7	13.9 ± 0.2	32.5 ± 0.1
*High PCO*_*2*_*+ Hypoxia*						
Rockfish	7.46 ± 0.01	1736 ± 58	4.7 ± 0.1	2237.2 ± 12.9	13.9 ± 0.1	32.6 ± 0.1
Cabezon	7.46 ± 0.02	1750 ± 107	4.6 ± 0.1	2237.1 ± 12.9	14.0 ± 0.1	32.6 ± 0.1
Phase II (Behavior/Predation)						
*Ambient PCO*_*2*_*+ Normoxia*						
Rockfish	7.85 ± 0.01	647 ± 19	7.85 ± 0.04	2221.7 ± 19.9	15.2 ± 0.1	32.4 ± 0.1
Cabezon	7.73 ± 0.05	908 ± 122	7.52 ± 0.42	2220.5 ± 18.4	15.3 ± 0.1	32.4 ± 0.1
*Ambient PCO*_*2*_*+ Hypoxia*						
Rockfish	7.91 ± 0.01	566 ± 6	4.87 ± 0.54	2214.2 ± 16.4	15.2 ± 0.1	32.4 ± 0.2
Cabezon	7.83 ± 0.04	701 ± 71	4.87 ± 0.20	2212.5 ± 13.4	15.2 ± 0.1	32.5 ± 0.1
*High PCO*_*2*_*+ Normoxia*						
Rockfish	7.38 ± 0.03	2098 ± 170	7.99 ± 0.04	2220.8 ± 17.4	15.2 ± 0.1	32.5 ± 0.1
Cabezon	7.42 ± 0.09	1950 ± 399	7.90 ± 0.24	2219.9 ± 16.4	15.3 ± 0.1	32.5 ± 0.1
*High PCO*_*2*_*+ Hypoxia*						
Rockfish	7.51 ± 0.05	1508 ± 192	5.38 ± 0.48	2204.2 ± 84.3	15.3 ± 0.1	32.4 ± 0.1
Cabezon	7.46 ± 0.10	1761 ± 435	5.03 ± 0.63	2200.2 ± 80.9	15.3 ± 0.2	32.5 ± 0.1

### Enzyme activity assays

After 1 and 3 weeks of acclimation, three rockfish were sacrificed from each replicate tank (*n* = 9 per *P*CO_2_/DO treatment). One cabezon was sampled from each replicate only after 3 weeks in each exposure (*n* = 3 per *P*CO_2_/DO treatment). We euthanized fishes in tricaine methanesulfonate (MS-222), recorded length and weight to calculate body condition and isolated a section of skeletal muscle tissue that was immediately frozen in liquid nitrogen. Due to the small size of the rockfish (~3 cm), a fillet of muscle, containing white and red muscle was isolated for biochemical assays. Following the experiment, all fish samples were transported to the main campus of University of California Davis, Davis, CA for enzyme quantification. We measured ATP-generating enzyme activity of citrate synthase (CS), cytochrome c oxidase (COX) and lactate dehydrogenase (LDH) to determine if *P*CO_2_/DO treatments altered cellular metabolism and/or increased energetic costs. CS and COX are both enzymes involved in aerobic metabolism in mitochondria, whereas LDH found in the cytosol is an indicator of glycolytic anaerobic metabolism (Jayasundara *et al.*, 2013).

Sample preparation and assays were conducted as described in Davis *et al.* (2017). Briefly, while COX was assayed fresh, CS and LDH were assayed on frozen supernatants. Each assay sample was run in triplicate, background activity rates were determined (CS and LDH) and enzyme activity rates are presented as μmol min^−1^ g fresh muscle weight (FW)^−1^. All assays were conducted in a spectrophotometer (Synergy HT, BioTek, Winooski, VT, USA) at 14°C (temperature at the collection site) and 24°C, a 10-°C increase for later Q_10_ calculations. Temperature of 14°C was maintained using an Isotemp BOD refrigerator chamber (Fisher Scientific, Waltham, MA, USA).

COX activity was monitored by the oxidation of reduced cytochrome c (Sigma, St. Louis, MO, USA) by the decrease in absorbance at 550 nm. CS was measured by monitoring the increase in coenzyme A-SH and concomitant increase in absorbance at 415 nm using dithionitrobenzoic acid (DTNB). LDH activity was monitored by the conversion of NADH to NAD+ and associated reduction in absorbance at 340 nm. Additional metabolic metrics including COX:CS and LDH:CS ratios were calculated. The ratio of COX:CS was used to evaluate changes in mitochondrial plasticity and oxidative capacity (i.e. aerobic ATP-energy production or limitation) in response to high *P*CO_2_ and low DO (Strobel *et al.*, 2013), whereas LDH:CS was calculated to evaluate metabolic potential and efficiency (anaerobic and aerobic; Hamilton *et al.*, 2017).

### Rockfish behavior assays

Anti-predator responses including exploration and activity behaviors were assayed using modified methodology from Ferrari *et al.* (2011a). The main difference in methodology was Ferrari *et al.* (2011a) examined changes in fish anti-predator responses in the presence of food, whereas the current study did not. Here, each fish was assayed individually in one of six, static 38 l (50 cm length × 25 cm width × 30 cm height) experimental tanks that were filled to 15 cm water depth of fresh seawater (14°C and 8.0 mg l^−1^ DO). The six tanks were placed next to each other in a row and surrounded by black plastic sheeting to reduce experimenter influence. An airline tube fixed to the inside of each experimental tank was used to inject cues via three syringes handled from outside of the black plastic sheeting. One syringe had a control cue of 10 ml seawater collected from the arena, and a second contained 10 ml of alarm cue. Non-predator conspecific alarm cues have been shown to affect a large range of taxa (Ferrari *et al.*, 2010). The alarm cue was freshly prepared as in Cripps *et al.* (2011) before each assay round by sacrificing a non-experimental rockfish and dorsoventrally scoring it seven times. Care was taken to ensure no organs were lacerated, only skin and muscle. The scored fish was then held over a beaker and rinsed with 10 ml of seawater six times, leaving 60 ml of alarm cue. This was split into six 10-ml batches for each of the six arenas. The third syringe was filled with 20 ml of seawater from the fish’s arena before the trial began and was used to flush the airline tubing after injecting the control and alarm cues. At the beginning of the assay the test fish was netted out of its *P*CO_2_/DO treatment tank and placed into the experimental tank. The assay included three phases: (1) a 15-min acclimatization phase to reduce the effects of handling stress, during which the fish was free to swim throughout the experimental tank and get used to its surroundings, (2) a 10-min baseline behavior phase (i.e. seawater cue) and (3) a 10-min anti-predator behavior phase (i.e. alarm cue). At the start of the baseline behavior phase, 10-ml seawater cue was injected into the tank at 5 ml s^−1^ followed by 10 ml of flush water. The seawater cue was injected to get a baseline behavior for each *P*CO_2_/DO treatment and to compare alterations in behavior before and after the alarm cue. After the 10-min baseline phase, the prepared 10-ml conspecific alarm cue was injected at 5 ml s^−1^ followed by another 10 ml of flush water. At the end of the anti-predator phase, each fish was removed with a net and placed into a separate 38-l tank, each aerated with the appropriate *P*CO_2_/DO treatment gas, in preparation for the predator–prey trials. The experimental tanks were emptied and cleaned multiple times with fresh seawater and set up for the next trial. At both 1 and 3 weeks acclimation time points, six behavior trials (with six individuals in each trial) took place each day for 3 days (*n* = 9 per *P*CO_2_/DO treatment per day, and *n* = 27 per *P*CO_2_/DO treatment each week) allowing 216 fish to be recorded in total. New fish were used at each of the 1- and 3-week time points. For each assay trial, fish were run at random (with respect to treatment) and baseline and anti-predator behavior phases were recorded using camcorders (520 line resolution QC with on-board IR) mounted above each of the six experimental tanks.

A 5-min period of each of the baseline and anti-predator behavior phases was played back on a computer monitor and analyzed manually by graphically applying a 5 cm × 5 cm squared-grid (50 grid squares total) to each tank to quantify the location of the fish over time. The first two minutes following the cue introduction was not included in this 5-min period to help control for physical disturbances from cue introduction such as airline tubing movement and air bubbles, but also the cue took >1 min to disperse through the arena (observed with dye injections). Each grid square was marked every time the fish entered into it and from this information two parameters were calculated: (1) area explored, defined as the number of novel squares entered (each square could only be counted once), and (2) activity, defined as the total number of times a fish crossed a grid line. To assess where rockfish were active within the arena, space use was quantified as the frequency of visits to each of the 50 arena squares (5 cm × 5 cm per grid square). To visualize potential effects of *P*CO_2_ and DO on space use, the average visits within each grid was calculated and plotted for the baseline and antipredator phases at both 1 and 3 weeks acclimation time using the *akima* package in R for interpolation of gridded irregular spaced data (Akima *et al.*, 2015). To test for a difference in space use before and after the alarm cue, four post-experimental zones were assigned to the arena including zones nearest the cue (5 × 25 cm^2^), side-walls (5 × 40 cm^2^), centre (15 × 40 cm^2^) and the farthest distance from the cue (5 × 25 cm^2^) (see Supplementary Fig. S2 for zonation details).

### Predator–prey trials

Six rockfish from each *P*CO_2_/DO treatment were randomly selected (with respect to the previous behavior trial) and transferred to a 180-l predation arena (91 cm length × 46 cm width × 43 cm height, with 42 cm water depth), with the respective *P*CO_2_/DO treatment water, and with one side of the arena having a standard volume of green ribbon anchored to a plastic rack to simulate submerged aquatic vegetation (Keller and Brown, 2008). A single cabezon, fasted for 24 h, was transferred to an affixed predator cage within the top corner of the predation arena. Both rockfish and cabezon were allowed 1 h to acclimate to the arena, after which the predator cage was opened and the cabezon were allowed to enter the predation arena with the rockfish. Each arena was covered with a black netted screen allowing for light penetration but minimizing the external disturbances. Predation trials lasted 24 h after which the surviving rockfish were counted, and predation mortality rate was calculated (number of fish eaten/total) as in Ferrari *et al.* (2015). All surviving fish of both species were removed, euthanized in MS-222 and length and weight were recorded for body condition index calculations. One predation trial for each *P*CO_2_/DO treatment (*n* = 4 total trials per day) was conducted each day for three days (*n* = 3 replicates per *P*CO_2_/DO treatment and time point). Predation trials were conducted after 1 and 3 weeks in experimental Phase II.

### Statistical analyses

Statistical analyses were conducted in R (version 0.98.1103, R Development Core Team, 2013), with statistical significance set at *P* < 0.05. All datasets were examined graphically via residuals vs. fitted values, residuals vs. treatment and acclimation time, *Q*–*Q* plots for normal distributions, and residuals vs. day and tank to detect any effects of replicates.

A three-way analysis of variance (*ANOVA*) was used to evaluate the effects of elevated *P*CO_2_, decreased DO and acclimation time on cellular metabolism metrics (COX, CS, LDH, Q_10_s and enzyme ratios) in juvenile rockfish. Cabezon cellular metabolism metrics were only measured at a single time-point, and hence a two-way *ANOVA* was conducted to test the effect of *P*CO_2_ and DO level on enzyme activities and *Q*_10_ values. A post hoc Tukey’s test (‘*lsmeans*’ package, Lenth, 2016) was used to determine differences in enzyme activities between *P*CO_2_, DO treatments, acclimation time. To determine effects of *P*CO_2_, DO and acclimation time on fish body condition index a three-way *ANOVA* was conducted for rockfish (log transformed) and cabezon.

A three-way linear mixed effect model *(lmer*, *nlme* package, Pinheiro *et al.*, 2015) was used to test the effects of *P*CO_2_, DO and the cue (seawater or alarm cue) on area explored and activity. Each *lmer* model included fish identification for repeated measures accounting for individual differences while also testing for treatment effects. A separate model was conducted for each acclimation time-point (1 and 3 weeks). An *ANOVA* table was generated for each *lmer* model, and significant *F*-statistics were followed by a Tukey’s post hoc test (*lsmeans*) to determine specific differences in treatment, time and the behavioral response before and after the cue. To compare the change in space use (i.e. post-defined arena zones) after presentation of the alarm cue across *P*CO_2_ and DO levels, a generalized least squares (*gls*) model was conducted to incorporate heterogeneity within the treatments into the model using the variance structure ‘varIdent’ with arena zone and *P*CO_2_/DO treatment combination as the fixed factors (*nlme* package, Pinheiro *et al.*, 2015; Zuur *et al.*, 2009). An *ANOVA* was then run on the *gls* model, and post hoc Tukey’s test was used to detect differences between *P*CO_2_/DO treatment and the interaction between zones using the *lsmeans* package. A separate *gls* model was conducted for each acclimation time point (1 and 3 weeks).

Predation mortality rate (the proportion of fish consumed) was arcsine-transformed to normalize data and analyzed with a two-way *ANOVA* as in Ferrari *et al.* (2011b), followed by a post hoc TukeyHSD, with fixed factors being *P*CO_2_/DO treatment combination, and acclimation time. All fish (rockfish and cabezon) metrics were originally nested within their replicate tank (*n* = 3 per tank) and measurement day (*n* = 3) within the model; however, with no tank or day effect, the models were reduced to simplest form and fish were pooled for a single treatment group.

## Results

### Physiology

#### Body condition

Body condition (i.e. Fulton’s K) of juvenile rockfish incorporating wet mass (g) and standard length (cm) measurements throughout the experiment showed no significant effects of *P*CO_2_ (*F*_1,724_ = 1.10, *P* = 0.29), DO (*F*_1,724_ = 0.047, *P* = 0.83) or the interaction between *P*CO_2_ and DO (*F*_1,724_ = 0.06, *P* = 0.81). Rockfish body condition changed across acclimation time (*F*_2,724_ = 3.28, *P* = 0.04) such that a decrease in condition was observed at 1 week but recovered by 3 weeks, matching initial pre-experimental body condition (Supplementary Fig. S3). There were no interactions between time and *P*CO_2_ (*F*_2,724_ = 0.82, *P* = 0.44), time and DO (*F*_2,724_ = 1.19, *P* = 0.30), or a three-way interaction (*F*_2,724_ = 0.32, *P* = 0.73). Body condition of cabezon was not altered by *P*CO_2_ (*F*_1,724_ = 3.27, *P* = 0.07), DO (*F*_1,724_ = 0.001, *P* = 0.98) or acclimation time (*F*_2,724_ = 2.09, *P* = 0.13), and there were no signification interactions between any variables (*P* < 0.05). See Supplementary Fig. S4 for cabezon body condition.

#### Rockfish enzyme activity

**Figure 1:**
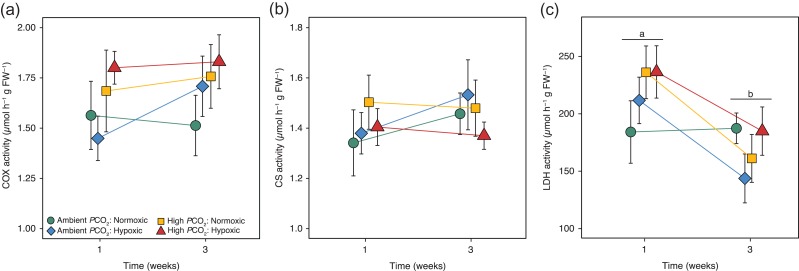
Aerobic and anaerobic metabolic enzyme activity in juvenile rockfish exposed to simulated CO_2_-acidification and hypoxia after 1 and 3 weeks of acclimation. Cytochrome c oxidase (**a**, COX), citrate synthase (**b**, CS), and lactate dehydrogenase (**c**, LDH) activity are presented as means (± SEM) for *n* = 9 fish per point. Coloured lines represent the enzyme trend across acclimation time in Ambient *P*CO_2_/Normoxic (green circle), Ambient *P*CO_2_/Hypoxic (blue diamond), High *P*CO_2_/Normoxic (yellow square), High *P*CO_2_/Hypoxic (red triangle). Lowercase letters in LDH activity, represent a significant difference by acclimation time (*P* < 0.05).

Aerobic metabolic enzyme activity COX and CS in juvenile rockfish was not significantly affected by *P*CO_2_, DO or acclimation time (Fig. 1a,b; see Table 2 for model results). Average COX activity was 1.67 ± 0.05 μmol min^−1^ g FW^−1^, whereas CS activity was 1.43 ± 0.05 μmol min^−1^ g FW^−1^. Anaerobic LDH activity significantly decreased across acclimation time (*F*_1,64_ = 9.88, *P* < 0.01, Fig. 1c), with no main effects of *P*CO_2_ (*F*_1,64_ = 2.27, *P* = 0.14), DO treatments (*F*_1,64_ = 0.02, *P* = 0.89) or interactions (*P* > 0.05, Table 2). Notably, under control conditions (Ambient *P*CO_2_/Normoxic), LDH activity was very similar after 1 week (184.14 ± 27.20) and 3 weeks (187.37 ± 13.31 μmol min^−1^ g FW^−1^) of acclimation time; whereas, in response to single and multiple stressor exposures of elevated *P*CO_2_ and/or hypoxia, LDH activity appeared to be elevated at 1 week and decreased roughly 32% (Hypoxia), 32% (High *P*CO_2_), and 23% (High *P*CO_2_/Hypoxia) after 3 weeks of acclimation (Fig. 1c).

**Table 2:** Models results for rockfish enzyme activity, ratios and *Q*_10_ values

Metric	Predictor	Enzyme activity				*Q*_10_		
		Df	SS	*F*	*P*	SS	*F*	*P*
COX	*P*CO_2_	1	0.607	3.168	0.080	0.239	0.729	0.396
	DO	1	0.045	0.237	0.628	0.297	0.906	0.345
	Time	1	0.127	0.664	0.418	0.1	0.304	0.583
	*P*CO_2_ x DO	1	0.037	0.193	0.662	0.017	0.051	0.822
	*P*CO_2_ x Time	1	0.022	0.113	0.738	0.023	0.071	0.790
	DO x Time	1	0.054	0.283	0.596	0.04	0.121	0.729
	*P*CO_2_ x DO x Time	1	0.107	0.561	0.457	0.404	1.231	0.271
	Residuals	63	12.064			20.685		

		Df	SS	*F*	*P*	SS	*F*	*P*
CS	*P*CO_2_	1	0.002	0.025	0.874	0.0006	0.055	0.815
	DO	1	0.01	0.108	0.743	0.0059	0.54	0.465
	Time	1	0.05	0.537	0.466	0.0154	1.414	0.239
	*P*CO_2_ x DO	1	0.117	1.253	0.267	0.0005	0.043	0.837
	*P*CO_2_ x Time	1	0.12	1.293	0.260	0.0183	1.682	0.199
	DO x Time	1	0.001	0.009	0.926	0	0.002	0.968
	*P*CO_2_ x DO x Time	1	0.003	0.027	0.869	0.0002	0.023	0.881
	Residuals	64	5.96			0.6949		

		Df	SS	*F*	*P*	SS	*F*	*P*
LDH	*P*CO_2_	1	9462	2.271	0.137	0.0109	1.204	0.277
	DO	1	72	0.017	0.896	0.0002	0.027	0.870
	Time	1	41 146	9.876	0.003*	0.0515	5.665	0.020*
	*P*CO_2_ x DO	1	1823	0.437	0.511	0.0001	0.015	0.904
	*P*CO_2_ x Time	1	4292	1.03	0.314	0.0004	0.04	0.842
	DO x Time	1	2560	0.614	0.436	0.0005	0.052	0.820
	*P*CO_2_ x DO x Time	1	10 061	2.415	0.125	0.0085	0.939	0.336
	Residuals	63	266 643			0.5818		

		Df	SS	*F*	*P*			
LDH:CS	*P*CO_2_	1	3903	2.609	0.111			
	DO	1	359	0.24	0.626			
	Time	1	23 413	15.652	<0.001*			
	*P*CO_2_ x DO	1	3548	2.372	0.128			
	*P*CO_2_ x Time	1	205	0.137	0.712			
	DO x Time	1	1914	1.279	0.262			
	*P*CO_2_ x DO x Time	1	5147	3.441	0.068			
	Residuals	64	95 736					
COX:CS	*P*CO_2_	1	0.374	5.404	0.023*			
	DO	1	0.007	0.1	0.753			
	Time	1	0.069	0.991	0.323			
	*P*CO_2_ x DO	1	0.397	5.74	0.020*			
	*P*CO_2_ x Time	1	0	0.002	0.964			
	DO x Time	1	0.073	1.06	0.307			
	*P*CO_2_ x DO x Time	1	0.148	2.14	0.148			
	Residuals	64	4.427					

Note: Asterisks indicate a significant (P < 0.05) effect of PCO_2_, DO, or Time on metabolic enzyme activity or ratio metrics.

**Figure 2:**
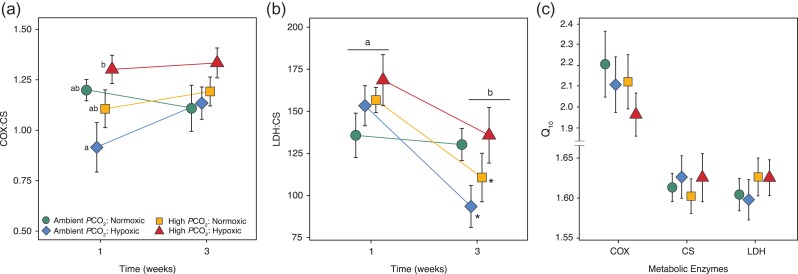
Metabolic potential and temperature sensitivity in juvenile rockfish exposed to CO_2_-acidified and hypoxic treatments. The ratios for mitochondrial change (**a**) COX:CS, metabolic potential (**b**) LDH:CS and (**c**) *Q*_10_ values for each enzyme are presented as means of *n *= 9 (± SEM). Coloured lines represent the enzyme trend across acclimation time in Ambient *P*CO_2_/Normoxic (green circle), Ambient *P*CO_2_/Hypoxic (blue diamond), High *P*CO_2_/Normoxic (yellow square) and High *P*CO_2_/Hypoxic (red triangle). Lowercase letters in (a) COX:CS represent a difference (*P* < 0.05) in *P*CO_2_ and DO treatments within 1 week, whereas lowercase letters in (b) LDH:CS represent an overall difference by acclimation time, with asterisks showing significant changes in ratios across time within a given treatment (*P* < 0.05).

The ratio of aerobic enzymes COX:CS was significantly altered by *P*CO_2_ (*F*_1,64_ = 5.40, *P* = 0.02), but the effect depended on the DO treatment (Normoxic or Hypoxic; *F*_3,64_ = 0.10, *P* = 0.75) with a significant interaction between *P*CO_2_ and DO (*F*_1,64_ = 5.74, *P* = 0.02, Fig. 2a). There was no effect of acclimation time or significant interactions between *P*CO_2_ and DO treatments and acclimation time (*P* > 0.05, Table 2). Under Normoxic conditions COX:CS ratios were most similar, independent of *P*CO_2_ treatment (*P* > 0.05); however, under Hypoxic conditions, the co-occurring High *P*CO_2_ significantly increased COX:CS ratios (*P* < 0.05) compared to the single stressor of hypoxia alone. LDH:CS (anaerobic to aerobic) was significantly altered by acclimation time (*F*_1,64_ = 15.65, *P* < 0.001), independent of *P*CO_2_ (*F*_1,64_ = 2.61, *P* = 0.11) and DO treatments (*F*_1,64_ = 0.24, *P* = 0.63) (Fig. 2b). While there were no two-way interactions between factors (*P* > 0.05, Table 2), a three-way interaction at *P* = 0.068, indicated the main effect of acclimation time had some dependence on *P*CO_2_ and DO treatment. For example, LDH:CS in fish exposed to single stressors (High *P*CO_2_ or Hypoxia) significantly decreased (*P* < 0.05) after 3 weeks of acclimation time compared to initial levels at 1 week, but this decrease was not significant in the multiple stressor LDH:CS remained similar to 1 week. Temperature sensitivity of enzymes quantified by Q_10_ values at assay temperatures of 14°C and 24°C showed only LDH was affected across acclimation time (*F*_1,64_ = 5.67, *P* = 0.02) with no effects of *P*CO_2_ or DO on Q_10_ values for COX, CS, and LDH (*P* > 0.05, see Table 2 for all model results). Average Q_10_ values for each *P*CO_2_/DO treatment are presented in Fig. 2c. Enzyme activity values measured at 24°C and *Q*_10_s for each acclimation time can be seen in Supplementary Tables S1 and S2.

#### Cabezon enzyme activity

Mean (± SEM) enzyme activities for cabezon predators at 3 weeks only are presented in Table 3. Overall, cellular metabolism in cabezon were unaffected by both single or multiple-stressors of CO_2_-acidifcation and hypoxia. Enzyme activities of COX, CS and LDH showed no effect of *P*CO_2_ or DO treatments (*P* > 0.05, see model results in Table 3). Furthermore, COX:CS or LDH:CS ratios were unaltered by *P*CO_2_ or DO treatments, nor Q_10_ values CS and LDH enzymes; however, *Q*_10_ values of COX were significantly decreased by elevated *P*CO_2_ (*P* < 0.05). All model results and interactions are presented in Table 3.

**Table 3:** Average (±SEM) enzyme activity metrics and *ANOVA* model summaries for cabezon acclimated to *P*CO_2_ and DO conditions for 3 weeks. Enzyme activities are expressed as μmol min^−1^ g FW^−^^1^, enzyme ratios of COX:CS and LDH:CS are given, and *Q*_10_ values from assays at 14°C and 24°C

Enzyme activity metric	Ambient *P*CO_2_ + Normoxia	Ambient *P*CO_2_ + Hypoxia	High *P*CO_2_ + Normoxia	High *P*CO_2_ + Hypoxia
COX	0.86 ± 0.13	1.01 ± 0.10	1.00 ± 0.24	1.08 ± 0.14
CS	1.01 ± 0.07	1.16 ± 0.09	1.06 ± 0.22	1.02 ± 0.10
LDH	85.77 ± 2.57	169.67 ± 20.17	85.32 ± 31.66	92.26 ± 14.81
COX:CS	0.84 ± 0.08	0.87 ± 0.03	0.93 ± 0.10	1.05 ± 0.07
LDH:CS	85.91 ± 4.98	126.91 ± 17.64	77.55 ± 15.04	91.13 ± 14.43
COX *Q*_10_	2.32 ± 0.16	2.29 ± 0.11	2.10 ± 0.14	1.89 ± 0.10
CS *Q*_10_	1.56 ± 0.07	1.59 ± 0.04	1.64 ± 0.01	1.62 ± 0.09
LDH *Q*_10_	1.83 ± 0.12	1.75 ± 0.04	1.85 ± 0.10	1.79 ± 0.08
*Model predictor*				
**Enzyme metric**	***P*****CO**_**2**_	**DO**	**Interaction**	
COX	*F*_1,9_ = 0.392, *P* = 0.547	*F*_1,9_ = 0.623, *P* = 0.450	*F*_1,9_ = 0.072, *P* = 0.795	
CS	*F*_1,9_ = 0.192, *P* = 0.672	*F*_1,9_ = 0.291, *P* = 0.603	*F*_1,9_ = 0.561, *P* = 0.473	
LDH	*F*_1,9_ = 2.094, *P* = 0.182	*F*_1,9_ = 2.600, *P* = 0.141	*F*_1,9_ = 1.498, *P* = 0.252	
COX:CS	*F*_1,9_ = 3.882, *P* = 0.080	*F*_1,9_ = 0.950, *P* = 0.355	*F*_1,9_ = 0.407, *P* = 0.539	
LDH:CS	*F*_1,9_ = 2.798, *P* = 0.129	*F*_1,9_ = 3.545, *P* = 0.092	*F*_1,9_ = 0.834, *P* = 0.385	
COX *Q*_10_	*F*_1,9_ = 6.070, *P* = 0.036*	*F*_1,9_ = 0.819, *P* = 0.389	*F*_1,9_ = 0.529, *P* = 0.486	
CS *Q*_10_	*F*_1,9_ = 0.757, *P* = 0.407	*F*_1,9_ = 0.023, *P* = 0.883	*F*_1,9_ = 0.288, *P* = 0.664	
LDH *Q*_10_	*F*_1,9_ = 0.191, *P* = 0.672	*F*_1,9_ = 0.631, *P* = 0.447	*F*_1,9_ = 0.008, *P* = 0.930	

Note: Asterisks indicate a significant effect (P < 0.05) of PCO2, DO, or an interaction between PCO2 and DO on enzyme metrics.

### Rockfish behavioral response

**Figure 3:**
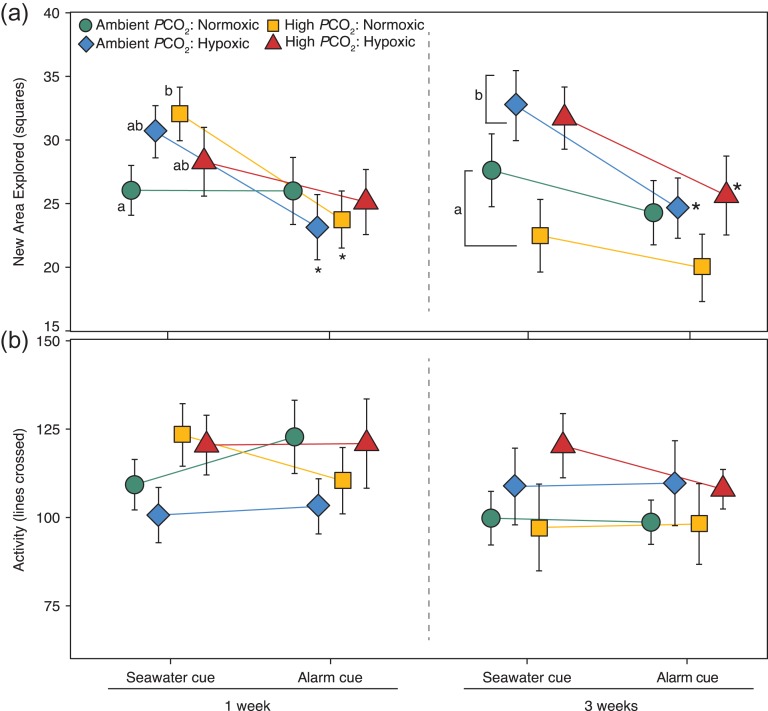
Response change of juvenile rockfish exposed to *P*CO_2_ and DO conditions for 1 and 3 weeks acclimation time. Mean ± SEM of (**a**) area explored and (**b**) total activity are shown after exposure to a seawater cue (baseline control) and a conspecific alarm cue (*n* = 17–24 per point). Coloured lines represent the behavior trend pre- and -post-alarm cue in Ambient *P*CO_2_/Normoxic (green circle), Ambient *P*CO_2_/Hypoxic (blue diamond), High *P*CO_2_/Normoxic (yellow square) and High *P*CO_2_/Hypoxic (red triangle). Lowercase letters indicate a significant difference in *P*CO_2_ and DO treatments within each cue, bracketed letters indicate a significant effect of DO level (Hypoxia) across cues, and asterisks signify a change in behavior between the seawater and alarm cue within a specific treatment (*P* < 0.05).

Area explored after 1 week of acclimation by juvenile rockfish was not altered by the main effects of *P*CO_2_ (Wald *X*^2^_(1,164)_ = 0.25, *P* = 0.62) or DO (Wald *X*^2^_(1,164)_ = 0.04, *P* = 0.84); however, area explored significantly decreased after the alarm cue (Wald *X*^2^_(1,164)_ = 18.82, *P* < 0.0001), with a significant 3-way interaction between *P*CO_2_, DO and cue (Wald *X*^2^_(1,164)_ = 8.87, *P* = 0.003). There were no significant two-way interactions between *P*CO_2_ and DO (Wald *X*^2^_(1,164)_ = 0.13, *P* = 0.72), *P*CO_2_ and cue (Wald *X*^2^_(1,164)_ = 0.75, *P* = 0.38), and DO and cue (Wald *X*^2^_(1,164)_ = 0.24, *P* = 0.62). Specifically, rockfish in the single stressor treatments (High *P*CO_2_ or Hypoxic) responded to the alarm cue by significantly decreasing the area explored (lsmeans Tukey, *P* < 0.05, Fig. 3a), whereas fish in control (Ambient *P*CO_2_/Normoxic) and multiple stressor (High *P*CO_2_/Hypoxic) conditions explored similar area in the seawater cue and alarm cue. During the baseline (i.e. seawater cue) phase, area explored by fish from the High *P*CO_2_/Normoxic treatment was significantly greater (*P* < 0.05) than those exposed to control conditions (Ambient *P*CO_2_/Normoxic); whereas after the alarm cue, area explored was similar in all *P*CO_2_/DO treatment groups (Fig. 3a).

Similar behavioral effects were observed after 3 weeks acclimation time (Fig. 3a), such that there was a significant decrease in the area explored following the alarm cue (Wald *X*^2^_(1,154)_ = 11.25, *P* < 0.001); however, after 3 weeks of acclimation, area explored was significantly affected by DO level (Wald *X*^2^_(1,154)_ = 5.12, *P* = 0.02), and not *P*CO_2_ (Wald *X*^2^_(1,154)_ = 1.13, *P* = 0.28). There was no significant two-way interactions between *P*CO_2_ and DO (Wald *X*^2^_(1,154)_ = 0.13, *P* = 0.72), *P*CO_2_ and cue (Wald *X*^2^_(1,154)_ = 0.75, *P* = 0.38) and DO and cue (Wald *X*^2^_(1,154)_ = 0.24, *P* = 0.62) or a three-way interactions (Wald *X*^2^_(1,154)_ = 0.24, *P* = 0.84). At 3 weeks, fish acclimated to Hypoxic conditions (single and multiple stressor) explored more area during the baseline seawater cue phase than fish acclimated to Normoxic conditions (*P* < 0.05). Additionally, the hypoxia-acclimated fish (single and multi-stressor) had a greater response to the alarm cue, decreasing the area explored compared to the seawater cue (*P* < 0.05).

Total activity, measured by the number of lines crossed yielded different results than exploration behavior (Fig. 3b). After 1 week of acclimation, activity behavior was not affected by *P*CO_2_ (Wald *X*^2^_(1,164)_ = 1.55, *P* = 0.21), DO (Wald *X*^2^_(1,164)_ = 0.42, *P* = 0.52) or the cue (seawater control and alarm cue; Wald *X*^2^_(1,164)_ = 0.07, *P* = 0.79). There were no significant interactions between *P*CO_2_, DO or the cue (*P* > 0.05). Activity after 3 weeks of acclimation was not affected by DO (Wald *X*^2^_(1,154)_ = 2.40, *P* = 0.12), *P*CO_2_ (Wald *X*^2^_(1,154)_ = 0.03, *P* = 0.86) or the cue (Wald *X*^2^_(1,154)_ = 0.66, *P* = 0.42), and there were no significant interactions between *P*CO_2_, DO or the cue (*P* > 0.05).

**Figure 4:**
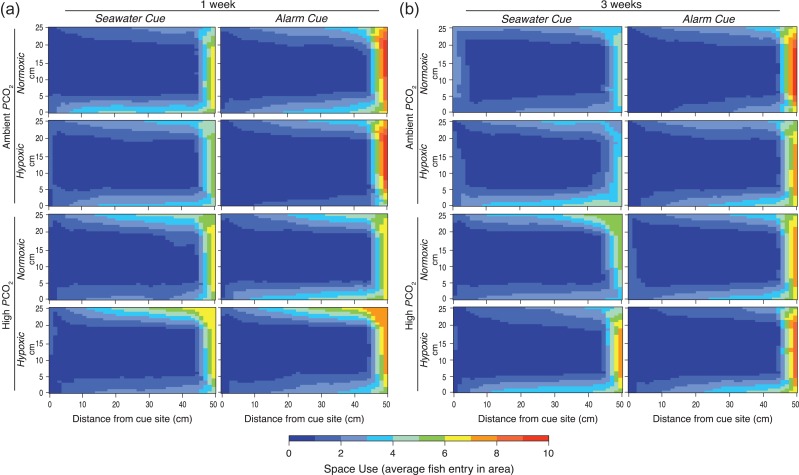
Two-dimensional aerial view of space use of rockfish. Data is presented as average activity within each 5 cm × 5 cm area (within the 25 cm × 50 cm arena) for each *P*CO_2_/DO treatment. Space use is separated by (**a**) 1 week and (**b**) 3 weeks acclimation time for the seawater cue (control) and after the conspecific alarm cue. Activity within the arena scales from no activity (blue = 0) to highest activity (red = 10).

Alterations in space use following an alarm cue were observed in rockfish after acclimation to *P*CO_2_ and DO treatments (Fig. 4). At 1 week of acclimation, a significant interaction (*F*_9,184_ = 7.47, *P* < 0.0001) between arena zones and *P*CO_2_/DO treatment combination indicated activity differed by arena zone (*F*_1,184_ = 15.73, *P* < 0.0001) depending on *P*CO_2_/DO treatment combination (*F*_3,184_ = 16.40, *P* < 0.0001). Rockfish under Ambient *P*CO_2_ (independent of DO level) increased activity in the zone farthest from the cue site, whereas rockfish under High *P*CO_2_, Normoxic and Hypoxic conditions showed little change in zone activity (lsmeans Tukey, *P* < 0.05, see zone assignments in Supplementary Fig. S2). After 3 weeks of acclimation, a change in space use following the alarm cue occurred in all four treatments (*F*_3,184_ = 8.51, *P* < 0.0001), characterized by a significant increase in activity in the zone (*F*_3,184_ = 92.65, *P* < 0.0001) farthest from the cue site. We detected significant trends in zone alterations, in particular increased preference for cells at the wall farthest from the cue site (Supplementary Table S3).

### Predator–prey trials

**Figure 5:**
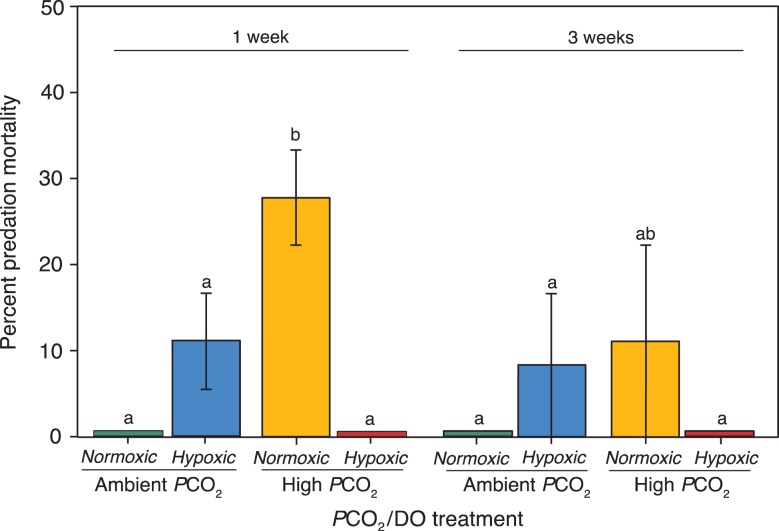
Mean (± SEM) percent predation mortality (proportion of rockfish consumed) of juvenile rockfish (*n* = 6) by cabezon predators (*n* = 1). Each bar is the average mortality rate of *n* = 3 predation replicate trials for each treatment: Ambient *P*CO_2_/Normoxic (green), Ambient *P*CO_2_/Hypoxic (blue), High *P*CO_2_/Normoxic (yellow) and High *P*CO_2_/Hypoxic (red). Lowercase letters indicate a significant difference in *P*CO_2_/DO treatments across 1 week and 3 weeks acclimation time (*P* < 0.05).

Predation mortality of rockfish was significantly altered by *P*CO_2_/DO treatment (*F*_3,15_ = 6.55, *P* = 0.005, Fig. 5), with no effect of acclimation time (*F*_1,15_ = 1.99, *P* = 0.18), or an interaction between *P*CO_2_/DO treatment and acclimation time (*F*_3,15_ = 1.26, *P* = 0.32). There was no predation mortality in the control (Ambient *P*CO_2_/Normoxic) and the multiple-stressor treatment (High *P*CO_2_/Hypoxic), but mortality increased to 28% and 12% at 1 and 3 weeks, respectively, in High *P*CO_2_/Normoxic (TukeyHSD, *P* < 0.05). A 12% increase in predation mortality was apparent only at 1 week in Ambient *P*CO_2_/Hypoxic (TukeyHSD, *P* = 0.12).

## Discussion

In this study, we characterized the interacting effects of CO_2_-acidification (high *P*CO_2_) and low DO (hypoxia) on cellular metabolism, behavior and predator–prey dynamics to assess whether rockfish living in seagrass beds have the ability to compensate physiologically or behaviorally to these naturally co-occurring stressors. Acute negative effects of elevated *P*CO_2_ and hypoxia were detected in juvenile rockfish after a one-week acclimation. Predation mortality increased with elevated *P*CO_2_ and hypoxia exposures, correlating with metabolic and behavioral adjustments. Following three weeks of acclimation, evidence of physiological and behavioral compensation was observed suggesting juvenile rockfish have some capacity to acclimate to elevated *P*CO_2_ and hypoxia. The observed resilience of juvenile rockfish may be due to their evolutionary history in an upwelling system, characterized by relatively frequent fluctuations in *P*CO_2_ and DO.

### Cellular metabolism

Rockfish showed trends of elevated metabolic costs under conditions of elevated *P*CO_2_ and hypoxia through increases in LDH activity, an index of cellular anaerobic capacity (Fig. 1c). In contrast, CS and cytochrome c oxidase (COX) activity, indices of cellular aerobic capacity, remained relatively unaffected. Similar metabolic adjustments have been observed in the spot, a common estuarine fish (*Leiostomus xanthurus*) exposed to hypoxic conditions (0.8, 2 and 4 mg l^−1^, Cooper *et al.*, 2002), and juvenile Antarctic rockcod (*Trematomus bernacchii*) exposed to high levels of *P*CO_2_ (~1200 μatm *P*CO_2_, Davis *et al.*, 2017) where increases in anaerobic LDH activity was observed, independent of changes in CS activity. Although non-significant, several trends of rockfish enzyme activity may warrant further discussion. Subtle elevations in LDH and COX enzyme activity in response to the multiple-stressor treatment were demonstrated by the highest mean enzyme activities from fish in the High *P*CO_2_ and low DO treatment at 1 week (Fig. 1a,c). These correlated increases in glycolytic (LDH) and respiratory chain activity (COX) may indicate cellular adjustments to maximize energy producing pathways and under the CO_2_-acidified and hypoxic conditions. Second, upward trends of COX and CS activities under hypoxic conditions (independent of *P*CO_2_ level) were demonstrated by an increase in mean activities from 1 to 3 weeks acclimation (Fig. 1a,b). Increased aerobic activity in response to hypoxia may indicate rockfish have increased the quantity of CS and/or COX enzymes to increase aerobic energy production under low oxygen conditions. In contrast to rockfish, cabezon predators in the present study exhibited no alterations in activity of any of the three metabolic enzymes (i.e. COX, CS, or LDH) after acclimation to single and multiple stressor treatments of elevated *P*CO_2_ and low DO (see Table 3).

Although CS activity in juvenile rockfish was unaffected by elevated *P*CO_2_ and hypoxia, functionally the ratio of CS to other key enzymes has been shown to be more revealing of metabolic adjustments and cellular reorganization (Windisch *et al.*, 2011). The synergistic interaction of increased *P*CO_2_ and hypoxia on rockfish enzyme ratios supports the hypothesis that the ability to cope with a single stressor may be compromised if combined with another stressor, an idea that is supported by previous work (see review Pörtner, 2008; Davis *et al.*, 2017). The highest mitochondrial (COX:CS) and glycolytic capacities (LDH:CS) were observed in rockfish under the multiple stressor treatment (high *P*CO_2_ and hypoxia) and suggest that metabolic costs were likely greatest under this exposure. Stress-induced metabolic costs could include acid-base regulation and protection mechanisms of proteins (Sokolova *et al.*, 2012, 2013). Potentially, in efforts to provide sufficient energy supply (i.e. cellular ATP production) for increased metabolic demands, mitochondrial and glycolytic adjustments were made in juvenile rockfish after 1 week of acclimation to treatment exposures. Metabolic adjustments by altered LDH:CS have been described in juvenile rockfish that inhabit kelp forests; however, in contrast to the present study, adjustments followed a single stressor of extremely high *P*CO_2_ (2800 μatm) and were observed after 5 months exposure (Hamilton *et al.*, 2017). The overall reestablishment of cellular energetic pathways after 3 weeks of acclimation with no obvious impacts on body condition (Supplementary Fig. S3) suggests that juvenile rockfish in the present study have the capacity to compensate if given sufficient time.

### Baseline behavioral alterations

The present study showed that absolute activity of rockfish was not affected by elevated *P*CO_2_ and hypoxia (Fig. 3b). This could indicate that there was sufficient aerobic capacity to support any elevated energetic demands caused by either of the stressors, such that activity was not compromised. Unaltered swimming activity in response to elevated *P*CO_2_ was similar to activity responses of number of other fishes exposed to high *P*CO_2_ including juvenile splitnose rockfish (1125 μatm, Hamilton *et al.*, 2014), juvenile spiny chromis (1000 μatm, Sundin *et al.*, 2017), anemonefishes (Nowicki *et al.*, 2011) and damselfish (Ferrari *et al.*, 2012), but contrasts with previous findings that showed significantly increased activity in juvenile coral trout (960 μatm *P*CO_2_, Munday *et al.*, 2013). Hamilton *et al.* (2017) showed negative effects of elevated *P*CO_2_ on rockfish performance (behavioral lateralization, Ucrit, and aerobic scope), but only at extreme *P*CO_2_ levels of >2000 μatm. Exploratory behavior in the current experiment, however, showed unique responses dependent on acclimation time and the interaction of DO level and *P*CO_2_ treatment. After a 1 week exposure, fish under elevated *P*CO_2_ (normoxia) explored more area compared to control fish, similar to exploratory and boldness behaviors documented in previous studies (Munday *et al.*, 2010; Ou *et al.*, 2015). It is possible that increased exploration was a result of elevated *P*CO_2_ reducing anxiety levels in fish (Hamilton *et al.*, 2014). The increased exploration under elevated *P*CO_2_ at 1 week was no longer present after 3 weeks such that exploration levels were more similar to the control. Therefore, there may be behavioral compensatory mechanisms that provide resilience to elevated *P*CO_2_ levels (Clements and Hunt, 2015; Duteil *et al.*, 2016). These rockfish may have adaptive mechanisms already in place to deal with high levels of *P*CO_2_ indicative of their natural seagrass bed environment, which exhibit large fluctuations on *P*CO_2_. After 3 weeks, fish exploration was most affected by hypoxia (independent of *P*CO_2_ level). Increased fish exploration under hypoxic conditions differed from our initial hypothesis that rockfish under hypoxic conditions would experience reduced activity and exploration behavior as an energetic conservation mechanism to reduce energy demand (Herbert and Steffensen, 2005). Increased exploration could be due to a motivation to escape low DO conditions (Herbert and Steffensen, 2005; Domenici *et al.*, 2000); however, it is important to note that behavioral assays were conducted in normal seawater (not *P*CO_2_/DO treatments), and hence, elevated exploratory behavior could have been a response to normoxic conditions.

### Anti-predator responses

The lack of change in fish activity following a conspecific alarm cue in any of the *P*CO_2_/hypoxic treatment conditions could suggest these juvenile rockfish lack the typical anti-predator response. Following a conspecific alarm cue it would be expected for prey fish to decrease activity in the presence of a predator (freezing behavior) or possibly rapidly increase movement (for improved predator confusion or evasion, Couzin and Laidre, 2009; Kelley *et al.*, 2011). In the current study, rockfish activity remained constant. In nature, these fish might respond to an alarm cue by actively seeking refuge (and then becoming inactive once in refuge), but the fact that they stayed active might reflect the lack of structural refuge in our test aquaria. While anti-predator responses were not evident in activity, exploratory behavior did reflect anti-predator responses. Rockfish exhibited a significant reduction in exploration when acclimated to single stressors of elevated *P*CO_2_ and hypoxic conditions, which contrasts with some previous work showing elevated *P*CO_2_ diminished predator recognition ability and thus prey maintained exploration levels after alarm cue exposure (Ferrari *et al.*, 2011a; Lönnstedt *et al.*, 2013). Importantly, however, fish exposed to a single stressor of elevated *P*CO_2_ or hypoxic conditions showed an altered response compared to control fish, consistent with the GABA hypothesis (Tresguerres and Hamilton, 2017). In the control treatment, the lack of a reduction in exploration following the alarm cue may be related to the fact that the conspecific alarm cue was injected after the baseline behavioral phase, and therefore was confounded with time spent in the arena. Fish are expected to decrease exploration with time, and rockfish had already explored area during the baseline phase in addition to a 15-min period provided after transferring the fish to the arena. However, it may also be the case, that rockfishes simply do not have a strong response to conspecific alarm cue.

The reduction in exploration following the alarm cue and the absence of any change in activity is better explained by looking at the space use analysis, detailing where the rockfish were active within the arena. Space use analyses showed there may be some short-term impairments to anti-predator responses after exposures to elevated *P*CO_2_ with fish under control treatments shifting the location of activity to areas farthest from the cue site (Fig. 4). This same shift in spatial activity (i.e. away from the cue site) was also seen in fish acclimated to hypoxic conditions. This spatial shift under hypoxia following the alarm cue further explains the reduction in exploration but not activity under hypoxia. While fish may have increased activity in only one section of the tank, the number of new squares they explored would naturally decrease. In contrast, rockfish in the high *P*CO_2_ (independent of DO treatment) after 1 week did not show a significant shift in space use following the alarm cue, which could be a result of impaired chemical detection mechanisms due to a malfunctioning of GABA_A_ receptors (reviewed in Tresguerres and Hamilton, 2017). After 3 weeks of acclimation, fish in high *P*CO_2_ showed a typical anti-predator response in spatial usage, as all treatment responses showed the expected pattern of an increase in activity in the area of the arena furthest from the alarm cue injection point. The return to a normal anti-predator response after 3 weeks of acclimation suggests rockfish have the capacity to compensate to some degree for elevated *P*CO_2_ if given adequate acclimation time. More research is warranted to uncover the mechanisms driving acute behavioral changes of rockfish in response to environmental stressors and whether elevated *P*CO_2_ interferes with brain ion-regulation, olfaction or vision shown in previous studies (Dixson *et al.*, 2010; Nilsson *et al.*, 2012, Heuer *et al.*, 2016; reviewed in Tresguerres and Hamilton, 2017). Assessments of behavior at finer spatial and temporal scales than traditional methods could allow for a more comprehensive understanding of how environmental stressors affect movement behavior (Ferrari *et al.*, 2011a; Duteil *et al.*, 2016).

### Predator–prey interactions

The effects of elevated *P*CO_2_ and hypoxia on predator–prey relationships appear to vary by species and are dependent on how both the prey and predator are affected (Ferrari *et al.*, 2011b; Couturier *et al.*, 2013; Midway *et al.*, 2017; Cripps *et al.*, 2011). The assessment of predator–prey relationships in the present study suggest prey (rockfish) and predators (cabezon) may be differentially affected by elevated *P*CO_2_ and hypoxic conditions. Rockfish under control conditions (Ambient *P*CO_2_/Normoxia) appeared to have adequate anti-predator escape responses after both 1 and 3 weeks of acclimation as none were consumed by cabezon (Fig. 5). Rockfish exposed to single stressors of elevated *P*CO_2_ and hypoxia experienced increased predation mortality after 1 week and 3 weeks; whereas rockfish predation mortality returned to zero under the multiple stressor treatment (High *P*CO_2_/Hypoxia). Cabezon stopped feeding on rockfish in the multiple stressor treatment (High *P*CO_2_/Hypoxia), suggesting cabezon were negatively impacted by exposure conditions. Indeed, other predators exposed to elevated *P*CO_2_ have displayed decreased activity (Rosa and Seibel, 2008), a reduction in their attraction to injured prey (Cripps *et al.*, 2011) and alterations in prey selectivity (Ferrari *et al.*, 2011b, 2015).

Our results provide evidence that the rockfish’s ability to evade predation under elevated *P*CO_2_ was decreased. Increased predation of rockfish under high *P*CO_2_ may indicate potential ‘trade-offs’ in physiological and behavioral strategies (Careau and Garland, 2012; Davis *et al.*, 2017). The significant predation mortality at 1 week under high *P*CO_2_ does correlate with the observed adjustments in cellular metabolism (i.e. reliance on anaerobic mechanisms) as well as the trends in the increased exploration and activity data, suggesting increased energetic costs under elevated *P*CO_2_ treatment. Furthermore, rockfish in high *P*CO_2_ showed no shift in space use in response to the alarm cue compared to the control suggesting potential sensory impairments (Dixson *et al.*, 2010; Nilsson *et al.*, 2012; Ferrari *et al.*, 2011a). These correlations in traits imply that physiological and behavioral trade-offs may have occurred, leaving rockfish less capable of avoiding predation. Metabolic and behavioral changes showed a degree of compensation after 3 weeks of acclimation, and predation mortality also decreased providing additional evidence these rockfish have the capacity to acclimate to elevated *P*CO_2_ over 3 weeks of acclimation.

In conclusion, juvenile rockfish and cabezon represent a valuable study system in which to explore the interaction of multiple changes in environmental factors over various biological scales. Predation pressure is very strong in seagrass communities (Hindell *et al.*, 2000), typically with high mortality of juvenile fish (relative to adults). Understanding the factors that determine juvenile rockfish survival during their time in seagrass beds is critical to understanding population dynamics. By combining physiology, behavior and predator–prey interactions this study described how three key interacting factors might be altered by elevated *P*CO_2_ and low DO, influencing juvenile rockfish survival. Juvenile rockfish had the capacity to acclimate to elevated *P*CO_2_ and low DO, such that short-term alterations in physiology and behavior were restored after 3 weeks of acclimation, which correlated with decreased predation mortality. Experiencing diel fluctuations in their natural seagrass bed environment, rockfish likely have adaptive strategies in place to cope with broad ranges of *P*CO_2_ and DO levels, as seen in a range of other species (e.g. blue rockfish [Hamilton *et al.*, 2017], shallow-shore crabs [Pane and Barry, 2007] and Pacific krill [Cooper *et al.*, 2016]). Cabezon predators showed no alterations of cellular metabolism following elevated *P*CO_2_ and low DO acclimation, but predation of rockfish ceased under multiple stressors indicating potential negative impacts on predator behavior. Studies that do not test both prey and predators under environmental stressors may misinterpret predator–prey interactions if differential species-specific sensitivities exist. Understanding how environmental stressors are integrated across biological scales, such as behavior and physiology in fishes, can be a powerful tool to identify trade-offs, assess vulnerability and project how community interactions may be altered under changing ocean conditions.

## Supplementary Material

Supplementary Data

## References

[coy038C1] Akima H, Gebhardt A, Petzold T, Maechler M (2015) Package akima: interpolation of irregularly and regularly spaced data. Package version 0: 5–12.

[coy038C2] Allan BJM, Domenici P, McCormick MI, Watson SA, Munday PL (2013) Elevated CO_2_ affects predator-prey interactions through altered performance. PLoS One 8: e58520. 23484032 10.1371/journal.pone.0058520 PMC3590170

[coy038C3] Appelhans Y, Thomsen J, Pansch C, Melzner F, Wahl M (2012) Sour times: seawater acidification effects on growth, feeding behaviour and acid–base status of *Asterias rubens* and *Carcinus maenas*. Mar Ecol Prog Ser 459: 85–98.

[coy038C4] Baumann H, Talmage SC, Gobler CJ (2012) Reduced early life growth and survival in a fish in direct response to increased carbon dioxide. Nat Clim Change 2: 38–40.

[coy038C5] Blaber SJM, Blaber TG (1980) Factors affecting the distribution of juvenile estuarine and inshore fish. J Fish Biol 17: 143–162.

[coy038C6] Brander KM (2007) Global fish production and climate change. Procs Natl Acad Sci 104: 19709–19714. 10.1073/pnas.0702059104 PMC2148362 18077405

[coy038C7] Briffa M, de la Haye K, Munday PL (2012) High CO_2_ and marine animal behavior: potential mechanisms and ecological consequences. Mar Poll Bull 64: 1519–1528. 10.1016/j.marpolbul.2012.05.032 22749063

[coy038C9] Careau V, Garland T Jr (2012) Performance, personality, and energetics: correlation, causation, and mechanism. Physiol Biochem Zool 85: 543–571. 23099454 10.1086/666970

[coy038C10] Caselle JE, Carr MH, Malone DP, Wilson JR, Wendt DE (2010a) Can we predict interannual and regional variation in delivery of pelagic juveniles to nearshore populations of rockfishes (genus *Sebastes*) using simple proxies of ocean conditions? Cal Coop Ocean Fish 51: 91–105.

[coy038C11] Caselle JE, Kinlan BP, Warner RR (2010b) Temporal and spatial scales of influence on nearshore fish settlement in the Southern California Bight. Bull Mar Sci 86: 355–385.

[coy038C12] Chan F, Barth JA, Lubchenco J, Kirincich A, Weeks H, Peterson WT, Menge BA (2008) Emergence of anoxia in the California Current large marine ecosystems. Science 319: 920. 18276882 10.1126/science.1149016

[coy038C13] Chivers DP, McCormick MI, Nilsson GE, Munday PL, Watson SA, Meekan MG, Mitchell MD, Corkill KC, Ferrari MCO (2014) Impaired learning of predators and lower prey survival under elevated CO_2_: a consequence of neurotransmitter interference. Global Change Biol 20: 515–522. 10.1111/gcb.12291 23765546

[coy038C14] Chung WS, Marshall NJ, Watson SA, Munday PL, Nilsson GE (2014) Ocean acidification slows retinal function in a damselfish through interference with GABA(A) receptors. J Exp Biol 217: 323–326. 24477607 10.1242/jeb.092478

[coy038C15] Clements JC, Hunt HL (2015) Marine animal behavior in a high CO_2_ ocean. Mar Ecol Prog Seri 536: 259–279.

[coy038C16] Cooper RU, Clough LM, Farwell MA, West TL (2002) Hypoxia-induced metabolic and antioxidant enzymatic activities in the estuarine fish *Leiostomus xanthurus*. J Exp Mar Biol Ecol 279: 1–20.

[coy038C17] Cooper HL, Potts DC, Paytan A (2016) Effects of elevated *p*CO_2_ on the survival, growth, and molting of the Pacific krill species, *Euphausia pacifica*. ICES J Mar Science 74: 1005–1012.

[coy038C18] Couturier CS, Stecyk JA, Rummer JL, Munday PL, Nilsson GE (2013) Species-specific effects of near-future CO_2_ on the respiratory performance of two tropical prey fish and their predator. Comp Biochem Physiol A Mol Integr Physiol 166: 482–489. 23916817 10.1016/j.cbpa.2013.07.025 PMC3830952

[coy038C19] Couzin ID, Laidre ME (2009) Fission–fusion populations. Curr Biol 19: R633–R635. 19674541 10.1016/j.cub.2009.05.034

[coy038C20] Crain CM, Kroeker K, Halpern BS (2008) Interactive and cumulative effects of multiple human stressors on marine systems. Ecol Lett 11: 1304–1315. 19046359 10.1111/j.1461-0248.2008.01253.x

[coy038C21] Cripps IL, Munday PL, McCormick MI (2011) Ocean acidification affects prey detection by a predatory reef fish. PLoS One 6: e22736. 21829497 10.1371/journal.pone.0022736 PMC3145675

[coy038C22] Davis BE, Flynn EE, Miller NA, Nelson FA, Fangue NA, Todgham AE (2017) Antarctic emerald rockcod have the capacity to compensate for warming when uncoupled from CO_2_-acidification. Global Change Biol 00: 1–16. 10.1111/gcb.13987 29155460

[coy038C23] Depasquale E, Baumann H, Gobler CJ (2015) Variation in early life stage vulnerability among Northwest Atlantic estuarine forage fish to ocean acidification and low oxygen. Mar Ecol Prog Ser 523: 145–156.

[coy038C24] Diaz RJ, Rosenberg R (2008) Spreading dead zones and consequences for marine ecosystems. Science 321: 926–929. 18703733 10.1126/science.1156401

[coy038C25] Diaz RJ, Breitburg DL (2009) The hypoxic environment In Richards J, Farrell AP, Brauner CJ, eds Fish Physiology, 27 Academic Press, Waltham, MA, pp 1–23.

[coy038C26] Dixson DL, Munday PL, Jones GP (2010) Ocean acidification disrupts the innate ability of fish to detect predator olfactory cues. Ecol Lett 13: 68–75. 19917053 10.1111/j.1461-0248.2009.01400.x

[coy038C27] Domenici P, Steffensen JF, Batty RS (2000) The effect of progressive hypoxia on swimming activity and schooling in Atlantic herring. J Fish Biol 57: 1526–1538.

[coy038C28] Doney SC, Ruckelshaus M, Duffy JE, Barry JP, Chan F, English CA, Galindo HM, Grebmeier JM, Hollowed AB, Knowlton N, et al (2011) Climate change impacts on marine ecosystems. Annu Rev Mar Sci 4: 11–37. 10.1146/annurev-marine-041911-111611 22457967

[coy038C29] Duteil M, Pope EC, Pérez-Escudero A, de Polavieja GG, Fürtbauer I, Brown MR, King AJ (2016) European sea bass show behavioral resilience to near-future ocean acidification. Royal Soc Open Sci 3: 160656. 10.1098/rsos.160656 PMC5180154 28018656

[coy038C30] Echeverria TW (1987) Thirty-four species of California Rockfishes maturity and seasonality of reproduction. Fish Bull 85: 229–250.

[coy038C31] Fangue NA, O’Donnell MJ, Sewell MA, Matson PG, MacPherson AC, Hofmann GE (2010) A laboratory-based, experimental system for the study of ocean acidification effects on marine invertebrate larvae. Limnol Oceanogr 8: 441–452.

[coy038C32] Feely RA, Sabine CL, Hernandez-Ayon JM, Ianson D, Hales B (2008) Evidence for upwelling of corrosive “acidified” water onto the continental shelf. Science 320: 1490–1492. 18497259 10.1126/science.1155676

[coy038C33] Ferrari MCO, Wisenden BD, Chivers DP (2010) Chemical ecology of predator–prey interactions in aquatic ecosystems: a review and prospectus. Can J Zool 88: 698–724.

[coy038C34] Ferrari MCO, Dixson DL, Munday PL, McCormick MI, Meekan MG, Sih A, Chivers DP (2011a) Intrageneric variation in antipredator responses of coral reef fishes affected by ocean acidification: implications for climate change projections on marine communities. Global Change Biol 17: 2980–2986.

[coy038C35] Ferrari MCO, McCormick MI, Munday PL, Meekan MG, Dixson DL, Lonnstedt Ö, Chivers DP (2011b) Putting prey and predator into the CO_2_ equation–qualitative and quantitative effects of ocean acidification on predator–prey interactions. Ecol Lett 14: 1143–1148. 21936880 10.1111/j.1461-0248.2011.01683.x

[coy038C36] Ferrari MCO, Manassa R, Dixson DL, Munday PL, McCormick MI, Meekan MG, Sih A, Chivers DP (2012) Effects of ocean acidification on learning in coral reef fishes. PLoS One 7: e31478. 22328936 10.1371/journal.pone.0031478 PMC3273466

[coy038C37] Ferrari MCO, Munday PL, Rummer JL, McCormick MI, Corkill K, Watson SA, Allan BJM, Meekan MG, Chivers DP (2015) Interactive effects of ocean acidification and rising sea temperatures alter predation rate and predator selectivity in reef fish communities. Global Change Biol 21: 1848–1855. 10.1111/gcb.12818 25430991

[coy038C38] Frieder CA, Nam SH, Martz TR, Levin LA (2012) High temporal and spatial variability of dissolved oxygen and pH in a nearshore California kelp forest. Biogeosciences 9: 3917–3930.

[coy038C39] Gattuso JP, Epitalon JM, Lavigne H, Orr J, Gentili B, Hofmann A, Proye A, Soetaert K, Rae J (2015) *Seacarb: seawater carbonate chemistry with R.* R package version 3.0.11. http://CRAN.R-project.org/package=seacarb.

[coy038C40] Gobler CJ, Baumann H (2016) Hypoxia and acidification in ocean ecosystems: coupled dynamics and effects on marine life. Biology Lett 12: 20150976. 10.1098/rsbl.2015.0976 PMC4892234 27146441

[coy038C41] Gobler CJ, Depasquale E, Griffith A, Baumann H (2014) Hypoxia and acidification have additive and synergistic negative effects on the growth, survival, and metamorphosis of early life stage bivalves. PLoS One 9: e83648. 24416169 10.1371/journal.pone.0083648 PMC3885513

[coy038C42] Hamilton SL, Logan CA, Fennie HW, Sogard SM, Barry JP, Makukhov AD, Tobosa LR, Boyer K, Lovera CF, Bernardi G (2017) Species-specific responses of juvenile rockfish to elevated *p*CO_2_: from behavior to genomics. PLoS One 12: e0169670. 28056071 10.1371/journal.pone.0169670 PMC5215853

[coy038C43] Hamilton TJ, Holcombe A, Tresguerres M (2014) CO_2_-induced ocean acidification increases anxiety in Rockfish via alteration of GABA_A_ receptor functioning. Proc R Soc B Biol Sci 281: 2013250. 10.1098/rspb.2013.2509 PMC3866405 24285203

[coy038C44] Herbert NA, Steffensen JF (2005) The response of Atlantic cod, *Gadus morhua*, to progressive hypoxia: fish swimming speed and physiological stress. Mar Biol 147: 1403–1412.

[coy038C45] Heuer RM, Grosell M (2014) Physiological impacts of elevated carbon dioxide and ocean acidification on fish. Am J Physiol Regul Integr Comp Physiol 307: R1061–R1084. 25163920 10.1152/ajpregu.00064.2014

[coy038C46] Heuer RM, Welch MJ, Rummer JL, Munday PL, Grosell M (2016) Altered brain ion gradients following compensation for elevated CO_2_ are linked to behavioural alterations in a coral reef fish. Sci Rep 6: 33216. 27620837 10.1038/srep33216 PMC5020430

[coy038C47] Hindell JS, Jenkins GP, Keough MJ (2000) Evaluating the impact of predation by fish on the assemblage structure of fishes associated with seagrass (*Heterozostera tasmanica*) (Martens ex Ascherson) den Hartog, and unvegetated sand habitats. J Exp Mar Biol Ecol 255: 153–174. 11108849 10.1016/s0022-0981(00)00289-6

[coy038C48] Hochachka P, Somero G (2002) Biochemical adaptation: mechanism and process in physiological evolution. Oxford University Press, New York.

[coy038C49] Hofmann GE, Barry JP, Edmunds PJ, Gates RD, Hutchins DA, Klinger T, Sewell MA (2010) The effect of ocean acidification on calcifying organisms in marine ecosystems: an organism-to-ecosystem perspective. Annu Rev Ecol Evol Syst 41: 127–147.

[coy038C50] Houston AI, McNamara JM, Hutchinson JMC (1993) General results concerning the trade-off between gaining energy and avoiding predation. Philos Trans R Soc Lond B Biol Sci 341: 375–397.

[coy038C51] IPCC (2013) Climate change 2013: The physical science basis. Contribution of working group I to the fifth assessment report of the intergovernmental panel on climate change. Cambridge University Press, Cambridge, UK.

[coy038C52] Ishimatsu A, Kikkawa T, Hayashi M, Lee KS, Kita J (2004) Effects of CO_2_ on marine fish: larvae and adults. J Oceanogr 60: 731–741.

[coy038C53] Ishimatsu A, Hayashi M, Kikkawa T (2008) Fishes in high-CO_2_, acidified oceans. Mar Ecology Prog Ser 373: 295–302.

[coy038C54] Jayasundara N, Healy TM, Somero GN (2013) Effects of temperature acclimation on cardiorespiratory performance of the Antarctic notothenioid *Trematomus bernacchii*. Polar Biol 36: 1047–1057.

[coy038C55] Jones MK, Mulligan T (2014) Juvenile rockfish recruitment in Trinidad Bay, California. Trans Am Fish Soc 143: 543–551.

[coy038C56] Kane AS, Salierno JD, Brewer SK (2005) Fish models in behavioral toxicology: automated techniques, updates and perspectives In Ostrander GK, ed Methods in aquatic toxicology. Lewis Publishers, Boca Raton, FL, pp 559–590.

[coy038C57] Keeling RF, Körtzinger A, Gruber N (2010) Ocean deoxygenation in a warming world. Annu Rev Mar Sci 2: 199–229. 10.1146/annurev.marine.010908.163855 21141663

[coy038C58] Keller K, Brown C (2008) Behavioral interactions between the introduced plague minnow *Gambusia holbrooki* and the vulnerable native Australian ornate rainbowfish *Rhadinocentrus ornatus*, under experimental conditions. J Fish Biol 73: 1714–1729.

[coy038C59] Kelley JL, Morrell LJ, Inskip C, Krause J, Croft DP (2011) Predation risk shapes social networks in fission–fusion populations. PLoS One 6: e24280. 21912627 10.1371/journal.pone.0024280 PMC3166168

[coy038C60] Kroeker KJ, Kordas RL, Crim RN, Singh GG (2010) Meta‐analysis reveals negative yet variable effects of ocean acidification on marine organisms. Ecol Lett 13: 1419–1434. 20958904 10.1111/j.1461-0248.2010.01518.x

[coy038C61] Laegdsgaard P, Johnson C (2001) Why do juvenile fish utilize mangrove habitats? J Exp Mar Biol Ecol 257: 229–253. 11245878 10.1016/s0022-0981(00)00331-2

[coy038C62] Largier J, Bickel A, Bahr F, Anderson D, Kudela R, Garcia-Reyes M, Nielson K, Vines K (2015) North Central-California MPAs: CeNCOOS Report on Environmental Conditions 2010–2015. In: North Central Coast State of the Region Assessment (2010–2015) Portfolio Product.

[coy038C63] Lenth RV (2016) Least-squares means: the R package lsmeans. J Stat Softw 69: 1–33.

[coy038C64] Lima SL, Dill LM (1990) Behavioral decisions made under the risk of predation: a review and prospectus. Can J Zool 68: 619–640.

[coy038C65] Lönnstedt OM, Munday PL, McCormick MI, Ferrari MC, Chivers DP (2013) Ocean acidification and responses to predators: can sensory redundancy reduce the apparent impacts of elevated CO_2_ on fish. Ecol Evol 3: 3565–3675. 24223291 10.1002/ece3.684 PMC3797500

[coy038C66] Li Z, Gray AK, Love MS, Asahida T, Gharrett AJ (2006) Phylogeny of members of the rockfish (*Sebastes*) subgenus *Pteropodus* and their relatives. Can J Zool 84: 527–536.

[coy038C67] Love MS, Yoklavich MM, Horsteinson L (2002) The Rockfishes of the Northeast Pacific. University of California Press, London, England.

[coy038C68] Midway SR, Hasler CT, Wagner T, Suski CD (2017) Predation of freshwater fish in environments with elevated carbon dioxide. Mar Freshwater Res. 68: 1585–1592.

[coy038C69] Miller SH, Breitburg DL, Burrell RB, Keppel AG (2016) Acidification increases sensitivity to hypoxia in important forage fishes. Mar Ecol Prog Ser 549: 1–8.

[coy038C70] Munday PL, Dixson DL, Donelson JM, Jones GP, Pratchett MS, Devitsina GV, Døving KB (2009) Ocean acidification impairs olfactory discrimination and homing ability of a marine fish. Proc Natl Acad Sci 106: 1848–52. 19188596 10.1073/pnas.0809996106 PMC2644126

[coy038C71] Munday PL, Dixson DL, McCormick MI, Meekan M, Ferrari MCO, Chivers DP (2010) Replenishment of fish populations is threatened by ocean acidification. Proc Natl Acad Sci 107: 12930–12934. 20615968 10.1073/pnas.1004519107 PMC2919925

[coy038C72] Munday PL, Pratchett MS, Dixson DL, Donelson JM, Endo GG, Reynolds AD, Knuckey R (2013) Elevated CO_2_ affects the behavior of an ecologically and economically important coral reef fish. Mar Biol 160: 2137–2144.

[coy038C73] Nagelkerken I, Munday PL (2016) Animal behaviour shapes the ecological effects of ocean acidification and warming: moving from individual to community‐level responses. Global Change Biol 22: 974–89. 10.1111/gcb.13167 26700211

[coy038C74] Nilsson GE, Dixson DL, Domenici P, McCormicj MI, Sørensen C, Watson SA, Munday PL (2012) Near-future carbon dioxide levels alter fish behavior by interfering with neurotransmitter function. Nat Clim Change 2: 201–204.

[coy038C75] Nowicki JP, Miller GM, Munday PL (2011) Interactive effects of elevated temperature and CO_2_ on foraging behavior of juvenile coral reef fish. J Exp Mar Biol Ecol 412: 46–51.

[coy038C76] Ou M, Hamilton TJ, Eom J, Lyall EM, Gallup J, Jiang A, Lee J, Close DA, Yun SS, Brauner CJ (2015) Responses of pink salmon to CO_2_-induced aquatic acidification. Nat Clim Change 5: 950–955.

[coy038C77] Pane EF, Barry JP (2007) Extracellular acid-base regulation during short-term hypercapnia is effective in a shallow-water crab, but ineffective in a deep-sea crab. Mar Ecol Prog Ser 334: 1–9.

[coy038C78] Pankhurst NW, Munday PL (2011) Effects of climate change on fish reproduction and early life history stages. Mar Freshwater Res 62: 1015–1026.

[coy038C79] Parker SJ, Berkeley SA, Golden JT, Gunderson DR, Heifetz J, Hixon MA, Larson E, Leaman BM, Love MS, Musick JA, et al (2000) Management of Pacific Rockfish. Fisheries 25: 22–29.

[coy038C80] Pichavant K, Person-Le-Ruyet J, Le Bayon N, Sévére A, Le Roux A, Quéméner L, Maxime V, Nonnotte G, Boeuf G (2000) Effects of hypoxia on growth and metabolism of juvenile turbot. Aquaculture 188: 103–114.

[coy038C81] Pinheiro J, Bates D, DebRoy S, Sarkar D, the R Development Core Team (2015) Package nlme: linear and nonlinear mixed effects models. R package version 3: 1–103.

[coy038C82] Pörtner HO, Lucassen M, Storch D (2005) Metabolic biochemistry: its role in thermal tolerance and in the capacities of physiological and ecological function. Fish Physiol 22: 79–154.

[coy038C83] Pörtner HO (2008) Ecosystem effects of ocean acidification in times of ocean warming: a physiologist’s view. Mar Ecol Prog Ser 373: 203–217.

[coy038C84] Pörtner HO, Peck MA (2010) Climate change effects on fishes and fisheries: towards a cause and-effect understanding. J Fish Biol 77: 1745–1799. 21078088 10.1111/j.1095-8649.2010.02783.x

[coy038C85] R Development Core Team (2013) R: A language and environment for statistical computing. Vienna, Austria: R Foundation for Statistical Computing. Retrieved from http://www.R-project.org/

[coy038C86] Rosa R, Seibel BA (2008) Synergistic effects of climate-related variables suggest future physiological impairment in a top oceanic predator. Proc Natl Acad Sci 105: 20776–20780. 19075232 10.1073/pnas.0806886105 PMC2634909

[coy038C87] Simpson SD, Munday PL, Wittenrich ML, Manassa R, Dixson DL, Gagliano M, Yan HY (2011) Ocean acidification erodes crucial auditory behavior in a marine fish. Biol Lett 7: 917–920. 21632617 10.1098/rsbl.2011.0293 PMC3210647

[coy038C88] Sokolova IM, Frederich M, Bagwe R, Lannig G, Sukhotin AA (2012) Energy homeostasis as an integrative tool for assessing limits of environmental stress tolerance in aquatic invertebrates. Mar Environ Res 79: 1–15. 22622075 10.1016/j.marenvres.2012.04.003

[coy038C89] Sokolova IM (2013) Energy-limited tolerance to stress as a conceptual framework to integrate the effects of multiple stressors. Integr Comp Biol 53: 597–608. 23615362 10.1093/icb/ict028

[coy038C90] Strobel A, Leo E, Pörtner HO, Mark FC (2013) Elevated temperature and PCO_2_ shift metabolic pathways in differentially oxidative tissues of *Notothenia rossii*. Comp Biochem Physiol B Biochem Mol Biol 166: 48–57. 23827663 10.1016/j.cbpb.2013.06.006

[coy038C91] Steckbauer A, Ramajo L, Hendriks IE, Fernandez M, Lagos N, Prado L, Duarte CM (2015) Synergistic effects of hypoxia and increasing CO_2_ on benthic invertebrates of the central Chilean coast. Front Mar Sci 2: 49.

[coy038C92] Sundin J, Amcoff M, Mateos-González F, Raby GD, Jutfelt F, Clark TD (2017) Long-term exposure to elevated carbon dioxide does not alter activity levels of a coral reef fish in response to predator chemical cues. Behav Ecol Sociobiol 71: 108. 28736477 10.1007/s00265-017-2337-x PMC5498585

[coy038C93] Taylor JC, Miller JM (2001) Physiological performance of juvenile southern flounder, *Paralichthys lethostigma* (Jordan and Gilbert, 1884), in chronic and episodic hypoxia. J Exp Mar Biol Ecol 258: 195–214. 11278010 10.1016/s0022-0981(01)00215-5

[coy038C94] Thorarensen H, Gústavsson A, Gunnarsson S, Árnason J, Steinarsson A, Björnsdóttir R, Imsland AK (2017) The effect of oxygen saturation on the growth and feed conversion of juvenile Atlantic cod (*Gadus morhua* L.). Aquaculture 475: 24–8.

[coy038C95] Todgham AE, Stillman JH (2013) Physiological responses to shifts in multiple environmental stressors: relevance in a changing world. Integr Comp Biol 53: 539–544. 23892371 10.1093/icb/ict086

[coy038C96] Torres JJ, Grigsby MD, Clarke ME (2012) Aerobic and anaerobic metabolism in oxygen minimum layer fishes: the role of alcohol dehydrogenase. J Exp Biol 215: 1905–1914. 22573769 10.1242/jeb.060236

[coy038C97] Tresguerres M, Hamilton TJ (2017) Acid–base physiology, neurobiology and behavior in relation to CO_2_-induced ocean acidification. J Exp Biol 220: 2136–2148. 28615486 10.1242/jeb.144113

[coy038C98] Wheeler SG, Anderson TW, Bell TW, Morgan SG, Hobbs JA (2017) Regional productivity predicts individual growth and recruitment of rockfishes in a northern California upwelling system. Limnol Oceanogr 62: 754–67.

[coy038C99] Windisch HS, Kathöver R, Pörtner HO, Frickenhaus S, Lucassen M (2011) Thermal acclimation in Antarctic fish: transcriptomic profiling of metabolic pathways. Am J Physiol Reg Integr Comp Physiol 301: R1453–R1466. 10.1152/ajpregu.00158.2011 21865546

[coy038C100] Wood CM, McDonald DG (1997) Global warming: implications for freshwater and marine fish. Cambridge University Press, Cambridge, UK.

[coy038C101] Zuur A, Leno EN, Walker N, Saveliev AA, Smith GM (2009) Mixed effects models and extensions in ecology with *R*. Springer, New York.

