# Supplementary material for: Juvenile rockfish show resilience to CO2-acidification and hypoxia across multiple biological scales
Source: Conserv Physiol. 2018 Jul 10;6(1):coy038. doi: 10.1093/conphys/coy038 (PMC6041801; doi:10.1093/conphys/coy038)
Supplement: Supplementary Data [file coy038_supplementary_material_conphys_06_09_2018.docx]

**Supplementary Material**

**Table S1.** Juvenile cabezon and rockfish enzyme activity values measured at assay temperature of 24ºC. Enzyme activities are expressed as μmol min^-1^ g FW^-1^, and asterisks indicate a significant effect of acclimation time (*P*<0.05).

| **Species and Enzyme** | **Time (week)** | **Ambient *P*CO_2_ + Normoxia** | **Ambient *P*CO_2_ + Hypoxia** | **High *P*CO_2_ + Normoxia** | **High *P*CO_2_ + Hypoxia** |
| --- | --- | --- | --- | --- | --- |
| Cabezon |  |  |  |  |  |
| COX | 3 | 1.94 ± 0.18 | 2.31 ± 0.21 | 2.06 ± 0.44 | 2.01 ± 0.17 |
| CS | 3 | 1.55 ± 0.05 | 1.84 ± 0.13 | 1.73 ± 0.36 | 1.67 ± 0.24 |
| LDH | 3 | 156.86 ± 11.57 | 257.93 ± 38.81 | 151.50 ± 46.59 | 167.58 ± 34.17 |
|  |  |  |  |  |  |
| Rockfish |  |  |  |  |  |
| COX | 1 | 3.09 ± 0.41 | 3.18 ± 0.26 | 3.62 ± 0.34 | 3.57 ± 0.34 |
|  | 3 | 3.42 ± 0.31 | 3.33 ± 0.34 | 3.42 ± 0.37 | 3.49 ± 0.26 |
| CS | 1 | 2.09 ± 0.23 | 2.28 ± 0.16 | 2.36 ± 0.18 | 2.23 ± 0.12 |
|  | 3 | 2.35 ± 0.13 | 2.47 ± 0.20 | 2.41 ± 0.71 | 2.27 ± 0.09 |
| LDH | 1* | 269.57 ± 38.38 | 347.22 ± 38.35 | 392.72 ± 38.82 | 387.45 ± 36.54 |
|  | 3 | 281.63 ± 16.81 | 223.82 ± 33.95 | 257.86 ± 34.45 | 298.49 ± 355.88 |

**Table S2**. Juvenile rockfish Q_10_ values from enzyme assays conducted at 14 and 24ºC, after 1 and 3 weeks acclimation time.

| **Species and Treatment** | **Week** | **COX Q_10_** | **CS Q_10_** | **LDH Q_10_** | **n** |
| --- | --- | --- | --- | --- | --- |
| Ambient *P*CO_2_ + Normoxia | 1 | 2.13 ± 0.23 | 1.61 ± 0.02 | 1.62 ± 0.03 | 9 |
|  | 3 | 2.28 ± 0.23 | 1.61 ± 0.03 | 1.59 ± 0.02 | 9 |
| Ambient *P*CO_2_ + Hypoxia | 1 | 2.24 ± 0.18 | 1.63 ± 0.03 | 1.64 ± 0.04 | 8 |
|  | 3 | 1.99 ± 0.19 | 1.62 ± 0.05 | 1.56 ± 0.03 | 9 |
| High *P*CO_2_ + Normoxia | 1 | 2.23 ± 0.12 | 1.57 ± 0.04 | 1.66 ± 0.04 | 9 |
|  | 3 | 2.02 ± 0.23 | 1.63 ± 0.02 | 1.59 ± 0.02 | 9 |
| High *P*CO_2_+ Hypoxia | 1 | 1.97 ± 0.15 | 1.59 ± 0.05 | 1.65 ± 0.03 | 9 |
|  | 3 | 1.96 ± 0.16 | 1.66 ± 0.04 | 1.60 ± 0.03 | 9 |

**Table S3.** Simplified trends of rockfish spatial usage following a conspecific-alarm cue. Spatial usage was significantly altered by *P*CO_2_/DO treatment (*P*<0.05), with specific zone changes indicated in this table. Trends were identified using a generalized least squares model, followed by a *lsmeans Tukey* test. + indicates an increase in activity and zone usage following the alarm cue, whereas a - indicates a decrease, and the = indicates no change in spatial usage from the initial seawater control cue. Letters indicate a significant difference in zone usage (*P*<0.05)

|  | **Ambient *P*CO_2_ + Normoxia** | **Ambient *P*CO_2_ + Hypoxia** | **High *P*CO_2_ + Normoxia** | **High *P*CO_2_ + Hypoxia** |
| --- | --- | --- | --- | --- |
| *1 week* |  |  |  |  |
| Nearest cue | **-** *a* | **-** *a* | **=** *a* | **-** *a* |
| Farthest cue | **+**  *b* | **+**  *b* | **=**  *a* | **=** *b* |
| Side walls | **=** *c* | **=** *c* | **=** *b* | **=** *a* |
| Center | **=** *c* | **=** *ac* | **=** *a* | **=** *a* |
| *3 week* |  |  |  |  |
| Nearest cue | **-** *a* | **-** *a* | **=** *a* | **=** *ac* |
| Farthest cue | **+**  *b* | **+**  *b* | **+**  *b* | **+**  *b* |
| Side walls | **=** *c* | **=** *c* | **=** *c* | **=** *c* |
| Center | **=** *c* | **=** *c* | **=** *c* | **=** *a* |

**Figure S1.** Conceptual diagram of carbon dioxide (CO_2_) and nitrogen (N_2_) gas delivery system used to maintain experimental treatments simulating CO_2_-acidification and hypoxia. Various units expressed are pressure per square inch (psi) and square cubic feet per hour (scfh).

**Figure S2.** Image of Zones applied in Space use analyses. Average rockfish length was 2.9 ± 0.25 cm. Letters represent ones of special concern whereas A) is the wall nearest the cue insertion, B) is the farthest distance (i.e. wall) from the cue, C) included both side walls, and D) the middle zone.

**Figure S3.** Body condition (i.e. Fulton’s K) of juvenile rockfish across time. Body condition was calculated as 100*(mass in g/standard length in cm^3^). The box represents the inter-quartile range (IQR) and the whiskers extend 1.5 times IQR. Within each *P*CO_2_/DO boxplot are individual fish data points for (a) Ambient *P*CO_2_/Normoxic (green), (b) High *P*CO_2_/Normoxic (yellow), (c) Ambient *P*CO_2_/Hypoxic (blue), and (d) High *P*CO_2_/Hypoxic (red) of body condition at time 0, 1 and 3 weeks of acclimation. Data points are from all experimental procedures, Phase I and II.

**Figure S4.** Body condition (i.e. Fulton’s K) of cabezon across time. Body condition was calculated as 100*(mass in g/standard length in cm^3^). The box represents the inter-quartile range (IQR) and the whiskers extend 1.5 times IQR. Within each *P*CO_2_/DO boxplot are individual fish data points for (a) Ambient *P*CO_2_/Normoxic (green), (b) High *P*CO_2_/Normoxic (yellow), (c) Ambient *P*CO_2_/Hypoxic (blue), and (d) High *P*CO_2_/Hypoxic (red) of body condition at time 0, 1 and 3 weeks of acclimation. Data points are from all experimental procedures, Phase I and II.
